# Supplementary material for: Postdocs’ advice on pursuing a research career in academia: A qualitative analysis of free-text survey responses
Source: PLoS One. 2021 May 6;16(5):e0250662. doi: 10.1371/journal.pone.0250662 (PMC8101926; doi:10.1371/journal.pone.0250662)
Supplement: S1 File — (DOCX) [file pone.0250662.s001.docx]

| 1 | 'Practice without science is blind, Science without practice is dead'. Do more research, be more practice |
| --- | --- |
| 2 | 1. Know thyself and count the cost. 2. Broaden your expectations, perspective and skill set so that they may be aligned with transferable skills that are valued outside of academia in case academia does not work out. |
| 3 | 1. Pursue your goals and work hard but have a backup plan. 2. In order to have a successful career in research you need to be three things: smart, hard-working and lucky. |
| 4 | 1) Choose the right lab (not only project, but also PI and talk to people from the lab first) 2) think about alternative for an academic career early 3) not everyone who publishes in the big three journals is a good scientist, and the other way around: if you don't manage to publish your paper there you can still be a good scientist. |
| 5 | A academic research career is very gratifying but it requires a lot of effort. But if it's the job you dream of, it's worth it. |
| 6 | A position in academia requires a clear individual drive and a purpose beyond thinking that it would be a neat and valuable role in society. A full academic position requires a high degree of luck on top of hard work as the statistics associated with getting a professorship are poor. Because of the time it takes to be successful, one should consider other directions if one has any doubt of success and a clear reason to pursue this course. |
| 7 | A successful career is only attainable for a percentage of those who enter into the field. The personal skills you will learn and the growth you will achieve is invaluable, but success is more dependent on politics and luck than the field would have you believe |
| 8 | a) Think about it end-to-end and talk to people in various stages of an academic career (PhD, postdoc, new and old faulty, possibly even people who have switched fields). b) Think about your skills and what makes you happy (independent of thinking about a career path). c) Take some time off and/or try a different path. A lot of people never leave academia and therefore often only see 'one way'. I recommend everyone takes some time away from the academic world to see how they like a non-academic life. |
| 9 | Ability. Aptitude. Give it a try through internship or volunteering and see if you have these two. |
| 10 | Academia isn't a job, it's a lifestyle |
| 11 | Academic research career is an excellent platform to continue your research |
| 12 | Academic research career is as hard as industry's one. You have to be very motivated, very competitive. And it can be very frustrating because sometimes no matter how much effort/ work you put in your work, success can just depend on your connections and who is willing to help you not how smart or dedicated you are. So motivation and passion are the keys... |
| 13 | Academic Research career is like sailing boat in rough sea...get ready for setbacks, paper rejections, grant rejections & get ready to handle difficult PI and lab-mate at times. |
| 14 | Academic Research career is very competitive on many levels: Great publication record Grant funding (very competitive) Strong faculty (PI) support |
| 15 | Academic research career requires dedication, patience, perseverance and an enormous amount of hard work for very long periods of time. Only if you are willing to do such long-term commitment, you should choose this as your career. |
| 16 | Academic research careers come at a cost of many other options. Consider why you want to conduct research and consider which aspects of research you like (writing, bench science, grantsmanship) |
| 17 | Academic research is great if you enjoy working hard, tolerate frustration, and accept that sometimes you need to work for years before seeing results. It's not that great if making money is important for you, if you need a lot of free time for your non-work life, and if you need frequent reinforcement and sense of success, |
| 18 | Academic research is mainly about solving scientific problems and critically analyzing research ideas. Develop your mind more than your hand. Read every day to criticize and understand not only to know. If you can not do it and enjoy it, get out. |
| 19 | Academic research is the most rewarding and most frustrating thing I've experienced. To be successful you need to know that there will be months of work that accumulate in nothing; no findings, no papers, no grants. Diagnostic labs and industry positions provide less flexibility but more straight forward results and conclusions. It's a game of priorities. |
| 20 | Advice I would give someone thinking about an academic research career is to try other options in science to know for certain if they really want to pursue an academic research career. |
| 21 | All career paths are horrible. Science is no exception, it is a truly heinous industry and many scientists should not be admired. But those egotistical and self interested people are everywhere and you have to learn to deal with them. To survive, it helps if you love the science. Truly passionate about the minute and technical details such that it is absorbed into your every day. It also really helps if your partner shares this passion. |
| 22 | Also consider other options outside academia. Unless really committed to academia, it is difficult to survive well in the academic world nowadays. |
| 23 | Always be excited about your research. Keep calm and do your best! |
| 24 | Always believe in yourself. Be persistent and confident. |
| 25 | Always have a back up plan, make sure to have collaborations in industry, make sure you have transferrable skills, do not only work on one project, but several, if you work on a risky project, get a second safer project to ensure you get regular publications, try to secure academic collaborations so that you can be on an authors list with less effort |
| 26 | Always keep thinking ahead so that your research is always moving forward. |
| 27 | An academic research career can be like being a gypsy. Jobs are temporary due to the nature of most of the grant funding programs. Your wife/husband career should be flexible to move from place to place. In the long term, owing a house results inconvenient if not imposible |
| 28 | An academic research career is no walk in the park. It takes years of hard work and high quality research to become a known person in the field. Then you also need the passion and perseverance to both teach others and write grants. |
| 29 | APPLY ACADEMIC KNOWLEDGE TO DISCOVER SOMETHING NEW |
| 30 | As trite as it sounds, follow your passion. It's too hard/impossible to succeed and be happy in this field (where money, family time and other earthly pressures can be extremely limited) without a strong internal drive and passion for what you do. |
| 31 | Ask for help. The greatest component to my success thus far has been asking the right people for help with grants, experiments, and other challenges. Without the support of my community it would be difficult to push forward research initiatives and secure funding for them. |
| 32 | Ask individuals that have the job you want what they like about their job and why they chose it. |
| 33 | Ask your mentors about the logistics, not just the research |
| 34 | Ask yourself if you're comfortable with a highly-competitive environment that sometimes feels more focused on securing grant funding than on anything else. Will you still be able to pursue your passions even given the challenging funding environment? How important is it for you to have free time outside of work and to be able to completely avoid checking emails and feeling accountable to work when not physically in your office? If you desire a career where you can work more of a '9-5' schedule and leave work AT work, an academic research career might not be for you. |
| 35 | Ask yourself whether you really enjoy research, considering its multiple dimensions. |
| 36 | At my most optimistic, I consider it like being a successful actor: the job you are envisioning (professor or, say, movie star) is very rare, and requires a lot of luck - it also requires a lot of hard work, but that is not sufficient. The job you will be more likely to get (researcher or, say, struggling theater actor) will only be satisfying if you truly love doing science above all else. At my realistic: you will be paid nothing and really not be making that big of a difference to the world (ex: as compared to medicine). You will have very view explicitly transferable skills and no one talks about other careers at all. At my pessimistic: Academia is a Ponzi scheme where the ones at the top get rich/successful and require an increasingly large number of students and postdocs to continue, those of whom have little chance of increasing their position in the food change. As a graduate student, you are like an indentured servant where your PI holds your PhD hostage until you do exactly what they want. Postdocs have a little more power, but you are paid WELL below market rates and are expected to work crazy hours. Regardless, my advice is always to not go into academia, period. Go to medical school, law school, whatever, but unless you are positive you want a very specific job that you know has a lot of openings and requires a PhD, you are making a very dumb decision. |
| 37 | Based on my some industry experiences, an academic job is much better than money seeking job because I believe innocent interest is much higher than any other job. |
| 38 | Be 100% committed. There is no room for doubt. |
| 39 | Be as versatile as possible. Do not train only for PI positions in academia. Make certain you devote time to develop skills for industry and/or non-bench careers. Get career advice from day one of the Ph.D. or before. Career development as a postdoc is more of an afterthought than anything really useful. |
| 40 | Be aware from the outset that it is not an easy road to take and to constantly be educating yourself in what other career options exist for you that will give you job satisfaction outside of academia in case it doesn't work out |
| 41 | be aware of all its aspects and be certain that it is the right choice for you. There are many other options available |
| 42 | Be aware of all options available to you. Although an academic career can be very rewarding and allows you to make great contributions, there may be other options that allow you to achieve your goals. |
| 43 | be aware of politics and the limited importance of meritocracy in academia. |
| 44 | Be aware of the extreme challenges and competitiveness and seriously consider other options as well. There are many jobs outside of academia that involve exciting scientific discovery. |
| 45 | be aware of the hard-work, endurance, and challenges that comes with it. be committed to scientific process and endeavor |
| 46 | Be aware that it is going to be less and less about the actual research as you move up. |
| 47 | Be aware that there will be long periods in which nothing positive happens. Be sure that you are passionate enough about the science to weather those spells. |
| 48 | Be careful and don't loose track of time. Don't make your happiness depend on your academic research career. |
| 49 | Be careful in your choice of mentor and project. Careers are made and broken on great and awful mentors. You can always try again in a postdoc, but, keep in mind, it's hard, and academic positions are few in number. |
| 50 | Be careful who you pick for a mentor. Ask around about this PI to see if he/she has an attitude that fits with your goals. Also, start researching career choices immediately upon starting graduate school and get involved in campus organizations to learn and network. Do not wait until close to graduation. |
| 51 | Be comfortable with failure and risk. Failing every day is normal and helps you learn. If you are not, you are not pushing the limits of your research and yourself . Riskier projects should be used to balance safe projects to move a field forward. This can open new areas of research for your own career and research program and enable you to publish in higher impact journals, which seems to be a requirement for obtaining a faculty position and succeeding. |
| 52 | Be curious, love learning, believe in yourself, people can have the right intentions as they guide you but be completely wrong, be flexible. |
| 53 | Be determined |
| 54 | Be driven by your hunger for reason, discovery and contribution to humanity. |
| 55 | Be efficient and focused, open minded but persistent, don't take critics personally and always give constructive feedback instead of 'critics', respect all unless proven otherwise (instead of the opposite). Collaborate when you can instead of competing. |
| 56 | Be focused and be innovative. Keep exploring current literature. |
| 57 | Be honest to yourself about your passion in science |
| 58 | Be honest with yourself about the climate of this career choice. This path is still relatively unforgiving for women who would like to have children and spend time with their family. Choose your projects and positions wisely. Ideally join a lab for a postdoc where you can contribute to a project that is wrapping up so you can have publications from the start of your postdoc. This will prevent a 'gap in productivity' that will hinder grant applications. |
| 59 | Be in a renowned lab. |
| 60 | Be in the top 5% or else you might love science, but science would not love you back. What I mean is unless you are exceptional, pursuing a career in science not worth it. In biomedical sciences, by 3rd year of your grad school you should know if you are cut out for this. Also, look for a financially stable partner, who has a real job, so that you can continue your fake postdoc job. |
| 61 | Be innovative in thinking. Work smart, not hard! |
| 62 | Be open minded and curious |
| 63 | Be open to other career paths that work better for the work/life balance |
| 64 | Be open-minded about viable alternatives |
| 65 | Be passionate |
| 66 | Be passionate about what you choose to study. |
| 67 | Be passionate with what you do and strive for continuous learning. You need to be patient as good results won't usually show up next day! Persistent effort and perseverance usually lead to rewarding outcome. |
| 68 | be passionate, be curious, be perfectionist, relieve your mind from other's judgment, know how to heal from setbacks |
| 69 | Be passionate, work hard |
| 70 | be patient |
| 71 | Be patient and keep on working hard |
| 72 | Be patient, optimistic, and persistent. |
| 73 | Be patient. |
| 74 | Be prepared and savvy about finding a postdoc position that will allow you to pursue and implement your research ideas, nurture your growth and independence. Establishing strong relationships with your colleagues and with collaborators is very important. |
| 75 | Be prepared for a lot of freedom in thought and ability to pursue academic interests with a great deal of effort in lab management, grant writing, and grant management |
| 76 | Be prepared for large sacrifices of time and slow progress. |
| 77 | Be prepared for lots of hard work, very little money, and lots of pressure. But in general, true scientists care very little about money or taking the easy route- they are just intellectually curious and looking for answers to their questions. |
| 78 | Be prepared for the long haul; this career path involves A LOT of time, patience, sacrifices and perseverance. There's a reason the percentage of Ph.D.'s is miniscule. |
| 79 | Be prepared to be persistent, have a thick skin |
| 80 | Be prepared to be working constantly. |
| 81 | Be prepared to earn a pittance and work overly long hours for a good 3-5 yrs as a postdoc with little job security. |
| 82 | Be prepared to give up your life and finances for the next 5-10 years. |
| 83 | Be prepared to not have a faculty academic position, but you can still be involved in academic research. |
| 84 | Be prepared to remain competitive and to seek out funding from alternative agencies, e.g. industry and foundations. |
| 85 | Be prepared to work constantly... |
| 86 | Be prepared to work hard |
| 87 | Be prepared to work hard - but know when to draw the line. There is a difference between working hard and being respected and working hard for no gain/appreciation/credit. |
| 88 | Be prepared to work hard and accept unsuccessful experimental results. Be tough. |
| 89 | Be prepared to work hard, but you also need to get lucky. |
| 90 | Be productive, write, and apply to as many grants as you can. |
| 91 | Be realistic about the current state of the job market. |
| 92 | Be realistic with your expectations, academia is just one possible career path, and isn't necessarily the most rewarding for you. Even if you do everything right, there's still a frustratingly low rate of getting a professorship. Don't sacrifice your happiness and sanity for that. |
| 93 | be resilient, failure is a part of learning both at the bench and securing funding |
| 94 | Be strategic with postdoc decisions. Think of it as getting training in an area that will enable you to secure independent training. Know and be willing to work long hours for a long number of years |
| 95 | Be sure about the career prospects, take time off after undergrad to work in industry and other areas. This will make you a stronger PhD student and help you make a career choice early on. You will have experience in both areas |
| 96 | Be sure that this is the type of career you enjoy. Otherwise, doing it will be difficult on a daily basis. |
| 97 | Be sure this what yoy want and that will make you happy and not because is the canonical pathway in research |
| 98 | Be sure to clarify what projects are going with you early on during the postdoctoral position. Develop those concepts and ideas well before entering the academic job market. |
| 99 | Be sure to love what you do |
| 100 | Be sure to take into account all the things that go into a research career. Talk to as many people as you can about their path and what they did to achieve success. Ask questions about what these people didn't realize or hadn't considered. |
| 101 | Be sure you are passionate about your field of student and are pursuing the career it for the right reasons, because it is not an easy career path. It allows for creativity, flexibility, and joys of learning and discovery, but is challenging in terms of funding, navigating bureaucracy and politics, administrative obligations, etc. There are other careers that are less demanding and more lucrative, so if you aren't passionate about your field of study, then think hard about if you want to pursue this career. |
| 102 | be sure you love it because it requires a lot of sacrifice and is not the easiest path |
| 103 | Be sure you really want the pressure. |
| 104 | Be sure you understand what it entails in addition to doing science - mentoring, writing grants and publications are a huge part of the academic life. |
| 105 | be sure you want an academic career |
| 106 | Be very careful in which labs and PIs you chose to work with. Check their previous projects, talk with previous lab members and take projects that you really love and are interesting for you |
| 107 | Be very sure that it is what you want because there is a lot more politics than there should be and you will have to play that game if you want to succeed |
| 108 | Be wary of pursuing such a competitive career |
| 109 | Be wise on the path you pick, however, anyone can be successful if they put their mind to it |
| 110 | Before entering into a career in academic research, consider your options at the end of your training. Are their job opportunities? Do the skills in your chosen field prepare you for a career other than academic research? It's best to choose a field of research that is not saturated with PhDs and can give job skills that are applicable in other backup careers. |
| 111 | Before starting an academic research career you should think about your values and what you want from your life. Pursuing an academic research career requires a lot of personal sacrifice. If you don't love science, or if what you do for a living is not the most important thing to you, then it probably isn't a good fit for you. |
| 112 | Before you plan for an academic research career, explore all the possibilities and think about what skills those careers require, what you are best at and what you most enjoy doing. |
| 113 | Begin with a PhD and see if academia would be a good fit for you. |
| 114 | Being a successful academic researcher is somewhat akin to pursuing a career in music performance or professional sports. Science and research must be your real passion for which you are willing to work extremely hard and sacrifice. And even with hard work and sacrifice, and of course the requisite level of talent, you may not make it to the big leagues. Be sure you are willing to take this risk and that you can enjoy the journey no matter what happens. |
| 115 | Believe in yourself and keep working hard. |
| 116 | Beware there are lots of politics. Be sure to select mentors in large labs that have publications in Nature, Cell, Science journals if you want an academic career. |
| 117 | Build your ability as early as possible. |
| 118 | Carefully consider the job and its requirements. |
| 119 | Carefully consider what you want in your career and get to know people at all stages of that career path to understand what their lives and trajectories are like. If you go down the academia road, carefully select your advisors (especially your postdoc advisor) for his/her connections and publication record based on the prestige/tier of the institution at which you desire a position. |
| 120 | carefully think about it, it will always be hard, never easy; you'll give most of your private life for that job but if you are feeling right in this career path, go for it |
| 121 | Chance is ~ same as getting a top job at Google. So, pretty much impossible. >99% of PhD students will have to find other option, so focus should be away from academic career! |
| 122 | change your mind |
| 123 | Check if you can really do a career in this specific fields and if grants are available for the field |
| 124 | Check the funding climate |
| 125 | Choose an excellent mentor at each level. Choose labs that have placed people in the type of careers you are interested in. |
| 126 | Choose carefully a lab you want to work as a postdoc. The research topics and ongoing studies in the lab should fit well on your interests, then you can enjoy doing your researches and finally get good publications for your next academic research career. |
| 127 | Choose the country and the university that can make this goal a real possibility. |
| 128 | Choose the right lab. Make sure it's someone well known, well funded, who has mentored people to academic positions before. This is so important when it comes to publishing in top tier papers l and getting K awards. |
| 129 | Choose this field only if you have an extreme passion in research and have wealth from family. |
| 130 | Choose well you're postdoc. Make sure you choose a lab with a good mentor that helps people and good track record |
| 131 | Choose wisely your labs and mentors. |
| 132 | Choose your mentor wisely. |
| 133 | Choose your mentors and collaborators wisely. Take advantage of every opportunity to network. |
| 134 | Choose your Ph.D advisor and post doc lab wisely. Position yourself early for success. Be reasonably sure/have good reasons for wanting to continue in academia |
| 135 | Choose your PI depending on how a good mentor he is more than how successful in the field he is. |
| 136 | Choose your post doc project wisely with a PI who will help you advance your career. |
| 137 | Choose your research environments carefully: make sure you are able to work on science that you are passionate about in a supportive environment. |
| 138 | collaborate |
| 139 | Collaborate, come up with innovative research questions and targets areas with good funding potential. |
| 140 | Commit only after you have investigated other options. But then, you have to really commit--sometimes to the point of putting aside other facets of your life--until you make it to where you are determined to be. |
| 141 | Consider academic career if you love doing the following: Science, Grant writing, Student advising, Working really really hard all your life for the above 3 reasons. |
| 142 | Consider all aspects of your work-life balance and how you like to work, what is important to you and what do you want to achieve by work, what is worth your effort. |
| 143 | Consider all possible career options -- if academia still tops your list, go for it; but definitely get all the information you can. |
| 144 | Consider all the extra responsibilities in addition to research- teaching, committees, writing grants, etc. |
| 145 | Consider carefully. Early stage pursuit of an academic research career usually involves moving cities just as soon as one becomes settled in one place and makes it difficult to really feel at home anywhere. I have found this, even with the support of a spouse, to be a somewhat isolating experience. |
| 146 | Consider how much you want to spend time on things outside of doing science experiments. |
| 147 | Consider mentors carefully. |
| 148 | Consider several option. Collaborate with others academic lab, get fund from private company. |
| 149 | Consider supply vs. demand |
| 150 | Consider the cost to benefit ratio. If you want financial security sooner than later, I would avoid science at least jobs in academia. Try to find jobs in academia that will provide good quality of life, assuming life outside of the lab matters to you, not just prestige. Consider alternative tracks than principal investigator, they are few and far between right now. |
| 151 | Consider the costs and benefits. The lifestyle is great (I get to do what I want, when I want), but it comes at a financial cost. At WCM, I feel undervalued and underpaid, but I am here because I like the people and it is a convenient geographic location for my personal circumstances. |
| 152 | Consider the funding environment. |
| 153 | Consider the number of times you are required to make major moves and how difficult that gets as one gets older. |
| 154 | Consider the price you pay on a personal level. If you are not flexible to relocate and move where your next job is, leave academia after grad school. If you have enough passion for science to get you through a decade of uncertain job prospects and long hours with little financial security it can be a rewarding career. Otherwise leave academia early. |
| 155 | Consider the work environment of the department and school choice. Gain insight through conversations with faculty, staff, or graduate students in the department of which you are choosing to work in. |
| 156 | Consider the years you will be paid poorly and the limited number of faculty positions. Consider doing PhD work that would be marketable to firms post graduation. |
| 157 | Consider what balance of work and personal time is acceptable and will make you happy. Those who reliably and regularly receive high impact publications and large grants tend to spend a majority of their time in lab writing, and have less free time outside lab. |
| 158 | Consider whether the process of obtaining an academic research career (getting a PhD, doing a postdoc, doing lab work every day) is enjoyable to you -- if it is, you should pursue it. If not, the outcome (i.e., a faculty job) is very scarce and will make the process downright unbearable. Therefore, I would only advise people to consider academic research careers if they would enjoy the journey, so to speak, not only the destination. |
| 159 | Consider why they want it, at what cost, and what they would be willing to comprimise on. |
| 160 | Consider your strengths and whether they are best utilized in leading a lab, which is ultimately what you will be doing ... the actual lab work will go away, and there is a big leadership component for which you most likely will not have received formal training but will be expected to do anyway. |
| 161 | Continually evaluate whether a research-intensive career is what you enjoy and are well-suited for. Be honest with yourself about your goals and expectations. Make yourself aware of other options for PhD level scientists. |
| 162 | Continue to network and get your research out (publish and attend conferences). |
| 163 | Culture a hobby out of the job, which can help you get your head clear, especially when you get frustrated from experimental failure. |
| 164 | Decide early if you want to pursue it and understand what it takes. Then go for it. |
| 165 | Decide the type of life they want and think hard about the reasons for doing such |
| 166 | Deeply think about how much where you work means to you. Also consider how much an academic position means to you versus an interesting industry career (think years and dollars). It is very satisfying, but costly, to pursue an academic position. Academic positions are sparse across the country/world - if you are picky about where you will live, consider that and know that it may add years to your job search. Think about this carefully long before starting your postdoc. Write down a list of what you want to get out of a postdoc and refer to this occasionally to make sure you are getting what you want out of your position. |
| 167 | determination |
| 168 | Develop a good sense of perseverance. |
| 169 | Develop a good understanding of the relevant job market as early as possible. |
| 170 | Develop a unique personal scientific 'brand' and spend most of your time working as lead author on projects that flesh out that brand. |
| 171 | Develop grant writing skills early in the postdoctoral fellowship. Spend time reading published research not just focusing on your own research. |
| 172 | Develop your brainstorming/project design skills. The opportunities to do that as a postdoc may be slim, but it is essential to being able to write your own grants and secure independent funding. |
| 173 | Discipline and enthusiasm are key! |
| 174 | Do anything else but pursue academic research career. The system is broken and it is too late to fix it. It is almost impossible to get grants. Postdocs are treated like slaves in many labs. It is extremely difficult to make ends meet on a postdoc salary and particularly if you are trying to raise a family. At the same time you are constantly being punished and penalized in research for having children. It is not a family friendly environment. |
| 175 | Do intensive research, not only about the area you're interested in, but the environment/morale of the lab itself before taking a job in a lab. |
| 176 | Do it because you enjoy the work that you do and not because you will be rich and famous. Do it to help move science forward without expecting anything in return. |
| 177 | Do it because you are driven to do it. It is incredibly difficult both intellectually and emotionally. |
| 178 | Do it because you are passionate about it. |
| 179 | Do it for love, not for money or power! |
| 180 | Do it for the passion, not the reward |
| 181 | Do it for the right reasons: your love of science. Because when nothing works and your PI is horrible, the only thing that will keep you going is your love for the science. |
| 182 | Do it if it's your one true passion, but there are easier ways to make money and make a difference. |
| 183 | Do it if you like doing it. |
| 184 | do it if you love to be it |
| 185 | Do it. |
| 186 | Do not base your sense of self-worth on having an academic position. Give it your best shot, but there are many viable pathways out there and you shouldn't feel that being an academic is the only viable option. |
| 187 | Do not be afraid to talk to your mentors with regards to acquiring funding and opportunities to communicate with others outside the lab. |
| 188 | Do not be bound by training. My degree is in biochemistry & molecular biology, yet I am now part of the pathology department. Be flexible enough to learn new skills, even when you think you are now an expert in your field. This will open up more doors and opportunities in a way you may not have predicted. |
| 189 | Do Not Do it. |
| 190 | Do not enter this field unless you have a driving passion for the work itself and for no other reasons. |
| 191 | do not expect to be fairly paid, do not expect the effort put in will be proportionate to success. |
| 192 | do not go if you are not fully committed |
| 193 | Do not go into it if you are not absolutely in love with your research field/ topic and are willing to trade financial security until much later in life. |
| 194 | Do not second guess your ideas just because they are different than the rest. That is precisely why we need to be at the table. |
| 195 | Do not take things personally, be persistent and use constructive criticism wisely. |
| 196 | Do not think of one. Seek other jobs as early as possible. Postdoc jobs are a trap. I wish someone told me that when I started |
| 197 | Do not underestimate the importance of work/life balance to be successful. It is often believed in science that because research is your passion, you must dedicate 110% of your daily resources. It is not true. Set the professional goals you are willing to achieve without endangering your personal life. |
| 198 | Do research into what industry jobs are available before you start; consider if your trajectory is compatible with academia; try to not get locked into a long-term postdoc position without hope of advancement. |
| 199 | Do something else. I am a successful young research scientist, but I would not advise a student to pursue a career in academic research. The balance between the effort put in, and the benefits gained is completely skewed. Dedication and hard work are no guarantee of success in terms publications. Very often, early career decisions as to the lab you apply to, to do your PhD training, have an inordinate influence in your career. Also, mentors have an inordinate influence in their students happiness and success, in some sort of warped and arcane master/serf dynamic that has somehow managed to survive in the present day, mostly unchecked by the indifferent academic institutions. Relative to their educational attainment and training, postdocs are very poorly paid, and there is little or no job security. There are so few faculty positions relative to the number of postdocs in academic research, that it is illogical to pursue a faculty position. Sadly, many academics don't fully realize this until well into their PhD, or perhaps they optimistically believe that they will be both good enough and lucky enough, to land a faculty position. Even when one acquires a faculty position, the intensive, competitive nature of academic research sees many PIs fail to make a meaningful impact in their fields. Academic research is broken. Whilst it is still a noble and worthwhile pursuit, why would anyone take a gamble with their careers, on a profession where the odds are very much against you reaching the top, and it is a certainty that as a postdoc, you will be payed peanuts and may not have a job in 6 months. Go do something where you will be appreciated for the smart and hard-working person you are. At this stage, only when young people turn their backs on academic research as a career choice, leading to the eventual negative impact on research outputs, will these issues be addressed. |
| 200 | Do something more lucrative from the start unless you know you REALLY want to be an academic scientific researcher. You can do good science in other types of jobs. Academia is not the end all be all, but if you wan to be a PI, commit 110%. |
| 201 | Do the best science you can do, surround yourself with people and mentors who will help you with all of the other things required to meet your goal. |
| 202 | Do what they enjoy; it may not be 'successful'. |
| 203 | Do what you love for as long as you can. There may come a point when it is no longer feasible (lost funding, not getting tenure, whatnot). But cross that bridge when it comes... Note: I should note that I have already secured a TT faculty position and will be starting shortly. The above is my thought process for embarking on this risky career path (given the current funding and political climate). |
| 204 | Do what you want and enjoy. |
| 205 | Do you really want this? |
| 206 | Do you truly enjoy research and the responsibilities (i.e. writing papers/grants) that come with those responsibilities? Are you competitive and confident in your ability to do science? Are you able to compartmentalize when research fails and not place blame on yourself? Are you not the primary caregiver in your family? If you answered yes to multiple of these questions, then you should consider an academic career. If you answered no to many of these questions, you might want to consider an alternative career. |
| 207 | Do you want this for the right reasons? |
| 208 | Do your homework about what it takes. |
| 209 | Do your homework before hand. Look into many different institutions and labs that may interest you and reach out to people currently working there. Get ready for years of hard work and make sure you are committed to the grind. |
| 210 | Doing good science is slow and hard, and there are many times in research that it is easy to get discouraged - make sure you identify a way to reignite your passion for research so that you can overcome those times of frustration - we need more people and more diverse ideas in this profession, not fewer. |
| 211 | Doing if you are convinced to want it because it is a lot of work ! |
| 212 | don't |
| 213 | Don't |
| 214 | Don't |
| 215 | Don't |
| 216 | Don't afraid to have data not expected. I'll give us better idea. |
| 217 | Don't be afraid of failure. |
| 218 | don't be naive. critically evaluate how you stack up against your peers. |
| 219 | Don't cheat to get there |
| 220 | Don't come for financial security. |
| 221 | Don't come to US if you are foreign, unless you are conscious that you will have to constantly fight for getting, renewing visas, and that you can be the most wonderful scientist in the world, but US immigration rules will not care about it |
| 222 | Don't despair - there is a random element in the job search process, so keep in mind that you may be perfectly qualified but bad luck happens. Keep trying! |
| 223 | Don't do it |
| 224 | Don't do it |
| 225 | Don't do it because it's a logical next step, but do it because you love your work. |
| 226 | Don't do it because of the money, science must be your passion |
| 227 | Don't do it for the money. Money will come and go. |
| 228 | Don't do it unless you are ok with having no life outside of research. |
| 229 | Don't do it unless you can't think of anything else you can do with your life. |
| 230 | Don't do it unless you really like research by itself. |
| 231 | Don't do it, it's a trap |
| 232 | Don't do it! |
| 233 | Don't do it! |
| 234 | don't do it! |
| 235 | Don't do it. |
| 236 | Don't do it. |
| 237 | Don't do it. |
| 238 | Don't do it. I tell every undergraduate student I mentor with an interest in science to pursue an MD or other career path vs pursuing a PhD. A career in science, especially academia, is the worst career path EVER. Everyone I know makes more money than me including my brother is 13 years younger and just graduated undergrad. I'm in my mid 30's and have worked 60+ hours a week for 10+ years for essentially minimum wage in hopes of getting an academic position. I don't own a house, drive a 15-year-old car, and my wife (also a scientist) and I have put off having kids when every else my age is comfortably settled with a family and mortgage. I just started to apply for tenure-track openings and have been told by dept. chairs that I need to have funding (K-award) in hand to be seriously considered for a position. I have 30+ publications and received my own funding since I was a graduate student (NIH F31 & F32 plus over $120K in supplemental funding). My K-award is currently under revision and feel my future is currently 100% dependent on my whether I get a K regardless of what is on my CV or my past accomplishments. |
| 239 | Don't do it. Or be ok with extreme and prolonged uncertainty. |
| 240 | don't expect to be parent of the year |
| 241 | Don't fucking do it. |
| 242 | Don't get a PhD unless you intend to complete research or work in think tank at the start of your career. Get your PhD funded including living costs and get your PhD at a large institution. This will increase your transferable skills. |
| 243 | Don't give up. |
| 244 | Don't give up. Stay focus on research and attendant more conference to upgrade your knowledge. |
| 245 | don't go for it |
| 246 | Don't go to the ivory tower there are far better and fair paying careers |
| 247 | Don't have a family... |
| 248 | Don't kill yourself over it, but don't give up if it is what you really want out of life. |
| 249 | Don't let anyone tell you what you cannot do. |
| 250 | Don't limit yourself to just academic career. Stay open minded and talk to people who've tried non-traditional career paths. |
| 251 | Don't listen to what researchers say unless they mention the existence of survivorship bias. Luck of the most important factor. |
| 252 | don't waste your time |
| 253 | Don't waste your time. There is not enough and there never will be funding!. Moreover, the system is rigged for the old established PIs to get grants over younger investigators. |
| 254 | Don't, if you have other attractive paths |
| 255 | Don't, unless you are very passionate. |
| 256 | don't, work will crush you |
| 257 | Don't!!!!! |
| 258 | Don't. |
| 259 | Don't. |
| 260 | Don't. |
| 261 | Don't. |
| 262 | Don't. Had I known what I know now, I'd have taken a different career route. |
| 263 | Don't. The funding situation gets worse every year and unless you find yourself in a hot field or high prestige lab, it's an uphill climb the whole way |
| 264 | Don#t think too big. Try to have a stable mid size Group and a stable and good outcome of publications |
| 265 | don`t have a family. |
| 266 | dont |
| 267 | Enhance time management skills, make good use of your time, and interpersonal skills are highly important as well. |
| 268 | Enter the field with open eyes to the challenges of obtaining Tenure Tract positions. There is little to no room for error in this ultra competitive environment. Success in academia is dependent on both hard work and a fair amount of luck and the single most important factor is strong mentorship. |
| 269 | Establish strong channels of communication with your PI early in your career (graduate or postdoc). |
| 270 | Establish strong mentors beyond your direct supervisor who will help with career development related areas. Take grant writing courses or workshops. Mentor students. Develop your own research questions and ideas as soon as possible in your career. |
| 271 | Everyone will tell you that such positions are impossible to secure and that the odds are very much against you. Try your best regardless. Have some back up career plans, but aim high anyway. |
| 272 | Explore all of your options, and make sure that you really are sure that you want to pursue that kind of career. |
| 273 | Explore other possibilities before committing to it. Know the pros and cons of a career in academia. |
| 274 | financial security is hard to establish, especially early on |
| 275 | Find a famous lab that publishes in high profile journals. |
| 276 | Find a field that you are genuinely passionate about. |
| 277 | Find a good mentor and publish solid papers to get cited |
| 278 | Find a good PI/supervisor - one of the most important steps to increase your chances of success. |
| 279 | Find a lab that has track record of postdocs transitioning to professorships. Your postdoc boss has the most influence on your own independent academic career. Its all about mentorship. |
| 280 | Find a lab with a PI that has a strong record training postdocs. |
| 281 | Find a mentor that likes you and helps you with your career goals in a timely manner! Otherwise, change your goals ASAP. Research is the worst field for capable people, unless your mentor is smart and compassionate. |
| 282 | Find a mentor who has the time and passion to see your grow and is willing to explain what the different career trajectories are, how you go about finding them, and wants to assist you in that journey. You need to find a mentor who values your potential as a future scientist and doesn't just view you as cheap labor. |
| 283 | find a nice PI that can give you scientific guidance and train your critical thinking at Ph.D. Then at postdoc, find a super big professor to accumulate papers and connections. Find the best timeline from the BEGINNING of the postdoc, including when to start applying for fellowship, when to start first draft of the transitional grant, and how long do you have to accumulate prelim data. 5-6 years sounds like a long time but is actually very tight if you plan to apply for a transitional grant to become a professor. |
| 284 | Find a place where you feel you would enjoy working, don't just go for publication background. |
| 285 | Find a very supportive mentor |
| 286 | Find a way to guest lecture or teach a course to see if it is something they would enjoy. |
| 287 | Find an environment that provides mentorship and support. Find an environment that values your well being. Find an environment where bragging about lack of rest/sleep is not the norm, but instead talking about what non-work related activity, personal well-being etc is encouraged. Find an environment that is collaborative with colleagues (students, post-docs and PIs) looking forward to meeting and discussing ideas together. |
| 288 | Find lots of mentors in different stages/fields/areas. |
| 289 | Find mentors beyond your supervisor. Its key to get connected and get influence. |
| 290 | Find something else. |
| 291 | Find the people you want to work with and figure out a topic to collaborate on together - it's a much healthier approach than working with nasty people with overlapping research interests. |
| 292 | Find the resources available at the current institution. |
| 293 | Find the right mentors early in your career |
| 294 | Find the right PI for your PhD. It will determine the rest of your career |
| 295 | Find what excites you and go for it. If you can't find something you are passionate about, it is probably best to do something else. |
| 296 | Find what you love and go for it. Don't let others tell you what you need (high impact publications, grant funding, etc.) in order to be successful. Sure, these things will be helpful, but if you are really motivated, there are plenty of other roads to get to where you want to be. |
| 297 | Find your own niche |
| 298 | Find your passion and let it lead you to your area of research. If you don't love it, don't do it! You will fall short; everyone does. Surround yourself with a good team of mentors and mentees. |
| 299 | Finding work-life balance is hard. Make sure you are happy with all parts of your life, not just your research success. |
| 300 | First and foremost, make sure that you love what you are doing! |
| 301 | First criteria for selection should be motivation and 'gut sense'. Networking and establishing a communication with PI for next steps in career could be of immense help. Finally, have some idea about plan B. |
| 302 | First, be patient when searching an answer for questions in science, Second, be persistent when you find that idea, Third, be prepared for failures, and finally, don't be afraid to start again. |
| 303 | Focus on acquisition of skills, rather than a specific project that interests you. |
| 304 | Focus on the aspects of the training and career that you most enjoy, and if you no longer enjoy doing it then this is not the right career path for you. Don't worry about how everyone else is doing, just focus on what you want to pursue. |
| 305 | Focus, perseverance, work on a simple idea with the basic application. |
| 306 | Follow research only when you are highly passionate about it, else there are lot of other things to do |
| 307 | Follow what you want to achieve |
| 308 | Follow your heart and think about what is important to you. It can be a wonderful career but there are many alternatives. Also, there are many different ways to build an academic research career and everyone does it slightly differently. Do what works best for you |
| 309 | Follow your own heart and do things that you are interested in. |
| 310 | Follow your own heart and work hard. |
| 311 | For an academic career one needs to have a strong base in their field of interest and should be able to develop scientific temper and interest in young minds and also convey the message in simplest manner. More or less he or she should be versatile. |
| 312 | For grad school and postdoc chose labs in a where the PI is established in his field because this is the way to get published in high ranking journals and to get your grants approved, without which you cannot develop a long lasting career in Academia. Learn to use advanced techniques in your research to make yourself attractive for collaborations in your future hiring academic institution. |
| 313 | For most people, I would advise against it. The lack of job stability and competitive nature of research careers with the pressure to publish and obtain grant funding is not an ideal environment. |
| 314 | For those interested in exploring career opportunities as faculty members at research intensive institutions (R1), please be aware that pedigree plays a significant role in getting your foot in the door. In this day, where each position has a significant number of applicants and positions are more competitive, it may be worthwhile to explore alternative career opportunities if you did not study at well-known institutions or with well-known researchers. |
| 315 | Fucking don't. Or, know what you are getting into, and the opportunity costs associated with it, how many PhDs are produced and how few of them actually get faculty positions, and how poorly equipped to deal with that reality your PIs are. Have an endpoint in mind for your career. Live in the moment. Enjoy doing science, but be ready to walk away at any time. |
| 316 | Fully immerse yourself in the science, the scientific community, and ongoing matters in science outside of your specific research. If this doesn't satisfy or interest you, then an academic research career is not for you! |
| 317 | Gain experience in research as soon as you can if you are interested. Ask to join a research lab during your undergraduate degree. If you enjoy it, stick with it and then pursue a graduate degree. |
| 318 | Generate novel ideas and know how to focus and develop them |
| 319 | Generating publications and grants is a lifestyle, just as much as it is a career. Many people I've encountered in the academic research setting work after hours. |
| 320 | Get a lot of practice writing effective grants as a student and post doc. Network in and outside of your feild |
| 321 | Get a MS and not a PhD and never do a postdoc. There are barely any jobs in academia and chances are after 5+ years as a postdoc, you will have to look for a non-academic job that NO ONE has ever provided any guidance on and is looked down upon from from nearly every PI. |
| 322 | Get a PhD and a postdoc in the right places. Build a strong network. |
| 323 | Get a PI that will want to author publications with you in top journals. Don't consider yourself a failure if you leave academia. |
| 324 | Get a position as a tech or RA in a lab and really pay attention to the day to day life of the PI. If it's academic research is only something you're 'thinking about', consider alternatives like industry. It is increasingly difficult to secure funding and people without a great passion for research may not find it enjoyable. |
| 325 | Get all the facts clear |
| 326 | Get exposure early and try different things...don't just stick to 'cancer research' or a focal type of work when you are young. Rotate through different environments under different mentoring styles - this helped me to identify the type of environment for training where I was most likely to fail, then grow, then succeed and learn how to think about a scientific hypothesis. |
| 327 | Get great mentor ship and direction. Get a community that supports you. Talk a lot about your science |
| 328 | Get informed of what you need to get there and how that is changing |
| 329 | Get plenty of cross-training to expand your skillset. An academic research career may lead to a faculty position, but most PhD graduates do not work as faculty. It is extremely important to be aware of the other career tracks that are available to you as a highly trained scientist and make sure to get the additional soft-skills and career specific training that can make you an eligible candidate for other types of careers in case you decide to not pursue a faculty position. |
| 330 | Get the training from the lab where has the history of publishing top journals. Name of the school is not as important as the quality of the paper your candidate lab publishes. |
| 331 | Get used to being told no and knowing otherwise. Make a commitment to yourself that it is ok to change your mind and to pursue other careers. |
| 332 | Get used to writing grants often and the unpredictability of grant funding. Have thick skin when it comes to paper reviews and idea planning. Be ready to have jobs and careers balanced on your shoulders. Basically: Be sure that grant writing and mentorship are what you want to do and are what make you happy. If not, think of other careers available to you as research-intensive faculty positions are highly/extremely competitive and not for everyone. |
| 333 | Get your work, life, and balance down and really make sure academia is what you want before pursuing it. |
| 334 | Getting a academic career will be competitive. One needs to be patient. Don't mind doing many years of postdocs. |
| 335 | Give more chance |
| 336 | Given the current funding environment and oversupply of people with life science PhDs that has accumulated since the mid-1990s, it's highly unlikely (less than 10% chance) you'll be successful. |
| 337 | Go after it if you have a passion for it. |
| 338 | go after your passion |
| 339 | GO FOR IT IF YOU ARE PASSIONATE ABOUT SCIENTIFIC RESEARCH |
| 340 | Go for it with full force |
| 341 | Go for it, but explore other options too. At some point you have to decide how long to pursue that career for and whether it is worth it. Personally, after a 6-7year post doc, if I am not successful in getting a faculty position and establish my own lab, I will not pursue a second post doc, I will look for something else. |
| 342 | Go for it, it will be worth it. Focus on the many positive aspects of an academic research career (creativity, learning new techniques, diverse skills, woking in a team, free trips, independence) and try not to think too much about publishing, grants or comparing yourself to others. |
| 343 | Go for it! |
| 344 | Go for it. Plan ahead. |
| 345 | Go in with your eyes open. All jobs have pros and cons. Academic jobs require you to be vigilant with grant writing and may never offer you the opportunity for advancement you seek. There are a lot of PhDs out there, so if you want to make it you must set yourself apart. |
| 346 | Go in with your eyes open. Science isn't fair. There is not enough funding for everyone. Not being successful in grants or with high impact publications doesn't make you a bad scientist. You need to be realistic about your chances of progressing in academia, and about the lifestyle. It's not for everyone, but it can be a great career. |
| 347 | Go into finance |
| 348 | Go into industry immediately after securing a bachelor's degree. Higher degrees are a liability. |
| 349 | go to industry |
| 350 | Go to medical school instead |
| 351 | Go with your passion |
| 352 | Good luck |
| 353 | good luck |
| 354 | Good luck! |
| 355 | Good luck! Perseverance, luck and opportunity will dictate your future. It will difficultly be in your hands the whole power of success. |
| 356 | Good luck... |
| 357 | grant writing will take up a lot of your time, often with no return for your effort |
| 358 | Great! Find a few good mentors at different stages in their pursuit of an academic research career (postdoc; young professor; older professor; etc). Ask yourself why you want to pursue that career path; observe your mentors and others in the positions that you are interested in. Ask questions about what it's really like (e.g. where is their time really spent, researching or grant writing/committees/etc.; how is the position different from what they thought it would be; how is it similar, etc) As long as that's what you want, go for it! There are a lot of great, happy, successful people in academic research careers, you can be one too! |
| 359 | hard work and novel ideas are key to sucess |
| 360 | Hard work and talent are not enough. Many other factors are involved. |
| 361 | Hard work is necessary but not enough. You have to be smart and insightful. This may not be learnable. |
| 362 | Hard worker, stay in the same field easier to publish Good to big lab where its easy to make collaboration |
| 363 | Have a back-up plan. There are a lot of researchers producing PhD students and the job market is flooded with young researchers and not enough academic positions. |
| 364 | Have a backup plan |
| 365 | have a good plan |
| 366 | Have a plan. Update it regularly. |
| 367 | Have a strong mentor and support system at your institution because your institutional resources will be heavily counted toward successfully obtaining a grant. |
| 368 | have a strong passion and resilience to negative results, or think about a different career path |
| 369 | have an alternative plan |
| 370 | Have fun with Science - not many academic research careers available, to keep an open mind about your job prospects |
| 371 | Have other career plans as well |
| 372 | Have thick skin and be prepared to put your career before family for a long time (wait and have kids once you've progressed enough or be OK with others caring for them). |
| 373 | high impact factor publications and secured grants |
| 374 | Honestly evaluate whether or not you think your passion for research at the start of your career is sustainable over the years. |
| 375 | I am an early stage scientist and am not sure if I can give a strong advice; so far I would say 'follow your passion'. I was given this advice. I will discover if it's a valid one. Obviously, all successful scientists can allow themselves to say it but what about the disappointed ones? |
| 376 | I am jaded and you should probably ask another person. |
| 377 | I am not inclined to go down the path of academic research, hence I cannot give advice to anyone. Anyone who is performing leading edge science, who has published at least 4 first author papers (at least 1 in a top journal), who has applied for and has been granted a K99/R01, should absolutely go for it. |
| 378 | I am pretty cynical about an academic research career, and therefore generally refrain from giving advice to people about it. However, trying to be positive, I would say that an academic research career can be very rewarding, but requires an enormous amount of work and time, and the reality of the situation is it can be done, but you really want to want it. (This is advice I wish I had received before returning to school to get a PhD in biology with academia as my focus) |
| 379 | I believe anyone who pursues a research career has to be prepare for work in a high pressure environment that consumes your life. There is really no separation between your research and life. You must be prepared to sacrifice holidays and special occasions with your family. |
| 380 | I can understand why you would want to complete your PhD but for the love of God please do not entertain a postdoc. Academia is in a very bad way at the moment; even for something as 'hot' as genetics and the life sciences you have a miniscule chance of a tenure track position in a decent college. I find it difficult to accept i wasted two years of my life as a postdoc but I am so happy I realised it now rather than later. Put it this way, even if you are in a top Ivy League school you still need a mentor who will fight for you (i see my supervisor who is an MD/PhD every 4 weeks or so, left to troubleshoot alone), a lab that is well published in your field of study, multiple other postdocs all working in a synergistic way and all the while accepting you live on a 'maybe' with regards your future and getting paid poorly for it. I love the freedom of the lab and the choice to work on whatever I really want but that really translates to 'whatever is hot and whatever I can sell to a grant committee'. I thought i was going to be one of those who beat the odds - I had at every step up until this point. Do yourself a favour and simply google 'reasons to leave academia for industry'. Read as many articles as you can, close your laptop, think about what you've read and act - all those figures, statistics and talented people who have come before you are not wrong. |
| 381 | I do not know enough about it to give advice. |
| 382 | I don't think I know enough to give advice about it |
| 383 | I have noticed that people that are successful in an academic career usually know very early on that they want to take this path. Therefore they could focus all their effort into this path, thereby increasing efficiency and success outcome. |
| 384 | I really need to explore all options around you and understand if you are really passionate about science. Science requires a lot of sacrifice and you need to be 100% sure that this path is right for you. Don't force yourself if you feel you don't want to become a PI - there are plenty of other exciting science-related opportunities outside academia. If you are passionate self motivated, enthusiastic and innovative researcher - go for faculty position! |
| 385 | I strongly recommend them to strengthen their basics. |
| 386 | I suppose I would advise them to network early and be confident that this is their passion. It requires a lot of dedication and is journey that can be lengthy and rocky at times |
| 387 | I think it is very important to identify the most important values in order to make the right professional choices and also to start thinking about these values as soon as you start your Ph.D. Basically, I would advise answering the following question: what is the impact that you would like to make with your work and over who? |
| 388 | I think it requires much responsibility, academic curiosity, and ability of educator |
| 389 | I think the basic is my publications, then is my Enlish skills can decide my career development in US. |
| 390 | I think the PhD is still a worthy goal. But I've come to realize the faculty position isn't the be all and end all of the process. There are many other useful and creative and important ways to use a Phd degree. I think new generations are coming around to that: they embrace alternative careers and they are being trained for them in better ways as universities accept the fact that most of us don't attain the faculty job. And it's not a good fit for everyone- how many PIs who are bad mentors do you know or have heard of? That's another problem, I think there should be some managerial training for tenure track faculty. And if they don't train people well, maybe they shouldn't get tenure. Another thing is, if you get the Phd and you want to leave academia, make a plan for that career- don't get sucked into a postdoc when you dont want to become a PI. |
| 391 | I think where you go to grad school is really important. I think even with all the capabilities I made a wrong choice. So this is my most important suggestion. |
| 392 | I would advice that the person to keep in mind that sometimes being successful and getting those top publications can be luck. Therefore it is always good to keep you mind open to other positions in the science community or research that they are interested in. Always have a plan B as the job market for an academic research career is really competitive. Visit their institution's career development office. |
| 393 | I would advice them to rethink about the academic research career considering following points 1. Its a huge time commitment 2. No Social life. 3. 90% of the time you will have to face failure. 4. You get paid very less. If somebody is very passionate about research then only they should choose it as a career. |
| 394 | I would advise against it |
| 395 | I would advise anyone to do research into academic research. I believe one must really love research to make a career out of it. If possible, work as an intern. |
| 396 | I would advise them to make themselves marketable in industry or other related professions in case an academic research position does not work out. Also, to make sure they understand that it is a long path. |
| 397 | I would advise them to reconsider. I would aldo advise them toexplote non academic roles. |
| 398 | I would advise this person to be reflective about their successes and failures, about their strengths and weaknesses. I have found my academic path to get the largest push from internal, rather than external, support. Be confident in yourself, and work to improve yourself. |
| 399 | I would ask them above and beyond the research,where do you see yourself? What would most likely want to do if you could as a career. |
| 400 | I would ask them if they knew what they were getting into ... |
| 401 | I would be scared to give any advice about our field. |
| 402 | I would encourage them to always be working on marketable skills even during graduate school. Given the uncertain political and economic climate, academic positions are hard to come by. Thus, I would caution prospective PhD's to be open to where their career might take them. |
| 403 | I would not advise it. |
| 404 | I would not recommend to pursue an academic research career given how the system works nowadays |
| 405 | I would recommend that they try to be as independent as they can in their research ideas, strategies and grant writing (from Grad school and on). If they are successful at each of these, and their ideas are well perceived by their field, then they likely have a shot. It is important to think about what you bring to the field and what you want to teach others through your research and mentoring. |
| 406 | I would recommend that this person do a significant amount of research and determine if this is really the best fit. Only 9% of PhDs land careers in academia, so there is a small chance, but for the majority of PhDs, it is not realistic. Look at your values, your level of patience, and your commitment to always writing grants and decide if that is what you want. Most scientists love the science, not the grant writing. In my experience, academic research gets you away from the bench and into the classroom or office. And in the office, you are constantly worried about grants, publications, and tenure, supervising others at the bench and hoping that they can make your dream come true. |
| 407 | I would say that one needs to be really passionated about their work on science to afford leaving your personal life aside to prioritize your professional development. It is s an extremely competitive and low-paid kind of job that requires intensive and life-long commitment. Think well whether it is something that really moves you and fulfills you. If it does, work really hard and do the best you can, always aiming at the top. |
| 408 | I would say that the academic research career can be very rewarding, given supportive circumstances, such as a good mentor, career guidance, collaborative peers, etc. However, this career depends on securing grant funding, which in turn depends on the success of your experiments and on the whims of those reviewing grant proposals. Perseverance and strong, supportive relationships with your peers and mentors are required for success in academic research. |
| 409 | I would say that to pursue this career path, you must have a passion for what you are studying, perseverance, and have a strong network of mentors, peers, and collaborators. This is not the path for those seeking primarily financial reward or job stability, unless you get very very lucky. |
| 410 | I would suggest being very smart and careful when choosing the lab and your future PI. |
| 411 | I would suggest getting experience working on and writing grants early to see if it something that they enjoy and have a skill for, as this is important to continued success. |
| 412 | I would suggest that instead they consider going into science policy or running for office to work on restructuring the failing U.S. scientific enterprise. |
| 413 | I would tell him/her that he/she should be prepared to work really hard, to sacrifice a lot of things of his/her personal life if he/she wants to achieve something special and then be able to enjoy the fruit of his/her labor. |
| 414 | I would tell them it is extremely competitive and that pursuing science can also be done outside of academia. |
| 415 | I would tell them it isn't easy. People that are successful in the academic field are not in it for the money or fame. Chances are you won't become famous or rich, but you do have the potential to help countless amounts of people, and if you are passionate about research and helping others, you shouldn't let the troubles of funding or the woes of others deter you from your goals. |
| 416 | I would tell them that it is like winning a lottery: buying a ticket (=working hard) is not sufficient to win (=publishing in a top journal), although it is required to win! |
| 417 | I would tell them to talk with lots of people in different roles within the field that they are interested in and ask very frank questions about the nature of the employment (not their research). For example, how many hours a week they work, what their starting pay was, how much debt they incurred getting their degrees, how career advancement happens in their field, what sorts of positions are most popular, most desired, and most commonly attained. |
| 418 | I would warn them that there are few job opportunities and that once you get into the field, it is hard to think you are able to do anything outside of the field. |
| 419 | I wouldn't |
| 420 | I'd advice to be very hard-working, patient and determined about what he/she wants. |
| 421 | I'm not far enough along to feel like I have a lot of advice that is well-tested, but I think it is most important to pick problems that are extremely exciting to you, because if you love them that will come through when you write about them for funding. |
| 422 | If it fits your life style, go for it! |
| 423 | If it is what you want, go for it, but don't be blinded to other options |
| 424 | If it is your passion and all you have dreamt about , go forth and put in all you have got to get into a good lab that publishes well and get into a field that is in demand. Do you research. If you are unsure of research, you will probably not pursue it later anyway so get out early. Educate yourself about all other opportunities as a phd scientist. There is a lot you can do and the earlier you know about the options the earlier you can start preparing for those career tracks. Dont do a postdoc just coz you are unsure about your next steps. You need to start thinking long term from your first year as a graduate student. |
| 425 | If it was someone considering starting a Ph.D program, I would ask them first if they have any research experience, and if not encourage them to get some first before applying. If they do have experience I would ask why they want to get a Ph.D. Is it because they feel it is the 'next step' in their career? Or is it because they want to be a PI? In my experience, the vast majority of new Ph.D students start a Ph.D because it seems like the next step in a pre-determined path, but without much idea about what career they actually want. |
| 426 | If it's what you want, you should absolutely pursue it. Don't let the fact that it's difficult deter you. |
| 427 | If making discoveries on a day to day basis, big or small, is what you crave for then is the career. If you have the patience to go through failure or unexpected results to find something new and interpret it, be criticized without giving up and then prove your point then this the career. If you are curious enough to get to the truth, no matter what but at your own pace then this is the career. If at the end of the day it's the happiness of your team on a earning a grant or a published paper or the success of your mentees that counts more than the awards you receive than this is the career for you. |
| 428 | If the career in research is driven by passion, perseverance and the realization that it is a very tough field to be successful with fewer rewards then I would say go for it. However, financially it will take a blow as you get older, have dependents. Besides the intelligence and handwork, networking is very important to be known in the field. I have changed my topic of research from cancer to obesity to bone development. It does show my adaptability and skills in several areas of research but a grant review committee gave the opinion that I am not serious about research and that I am constantly shifting my goals. Researchers end up in different labs with different skill sets (publication rates, techniques, the lab's fame in a particular field are all important) and that can make or break one's career. If an academic research career is what the person needs then years of planning is necessary. it shouldn't be something that one should be thinking at the second year of postdoc. Publications are key. How much ever one argue that impact factors are irrelevant and true science will triumph, the world doesn't work that way. At the end of the day, impact factors and the prestige of the journals are considered as key factors to get a tenure. Doing research itself is difficult and getting published is more and more difficult these days because of the number of experimental data the reviewers demand some of which are outrageous. So, anyone who is thinking of an academic research career needs to be aware of all these. |
| 429 | If you absolutely love research then go for it. All of the struggle and obstacles getting there have to be worth it because It feels like your life is goin in slow motion while everyone around you is advancing and getting paid more and having more free time. |
| 430 | If you are absolutely passionate about it and there is nothing else that interests you, then you should do it. Science is important and needs good passionate people. But you're likely to fail to get a good/stable position and even if you do, it's going to be hard, frustrating, and not always fair. |
| 431 | If you are indeed interested in the science research, keep going on to pursue your dream. |
| 432 | If you are interested in it then be all in and go to the best supervisors at the best schools. However, be adaptive and aware of the current economic market. |
| 433 | If you are interested then only come to this field, as jobs are less and competition is high. |
| 434 | If you are looking for money with this option don't come. |
| 435 | If you are not ready to devote your life, don't do it. |
| 436 | If you are not willing to play dirty and use others to your advantage, then forget about succeeding in academic research career as a PI.. Unfortunately, this is my observation at UIC. |
| 437 | If you are passionate about it, go for it. However, look at the work-life balance, job security, travel commitment, and other aspects of the job before you choose it. Also, make sure you look into other careers to make sure you're making a fully educated decision. |
| 438 | If you are passionate about reserach and does not care about teaching, I think it is a very rewarding career. Just make sure that's what you want |
| 439 | If you are passionate about your research, and you are ok with putting your family, friends, and hobbies on the back-burner for the majority of your life (20's-50's), then it's probably a good fit for you. If you want to live a balanced lifestyle where you can do your 9-5, go home and have dinner with your family, have a garden, pick up a guitar and play every now and then, I wouldn't go into academia. Your priorities are not in line with those who make the standards in academia (elitist white males). |
| 440 | If you are passionated for science, go for it! |
| 441 | if you are really committed then only enter the STEM field |
| 442 | If you are really interested in, just dive-in. |
| 443 | If you can avoid doing research don't. Academia value only publications, not efforts. |
| 444 | If you can do anything else, do that instead. |
| 445 | If you can make a living and be happy doing anything else, do it (same advice I got about whether or not to choose surgery in medical school). I think an academic research career can be a wonderful way to spend your life, but only do it if you think about it in the shower. |
| 446 | If you can succeed in basic research, you can be more successful in industrial, financial or other field. If you insist, choose a big name (e.g., Nobel laureate, NAS member, HHMI, or at least a chairman of department) as your mentor. |
| 447 | If you can't deal with frustration don't start an academic research career |
| 448 | If you care about money, don't come. This field is sustained only by passion now. |
| 449 | If you don't want to be a PI, don't do a postdoc |
| 450 | If you feel passionate about it, go for it |
| 451 | If you have a passion for research, do it. If not, you may consider doing academic research for a short period of time to get you to the place where you really want to be. |
| 452 | If you have any doubts about it, don't go for it. |
| 453 | If you have any other hobbies, pursue them first. Academia isn't going anywhere. |
| 454 | If you have doubt in working hard to do science, it is not a good choice to follow academic research career. |
| 455 | If you like it, just to enjoy it! Don't think too much! |
| 456 | If you love it, love it to your heart. No matter what the price is |
| 457 | If you love it, make it happen. Focus on publishing papers |
| 458 | If you love it, pursue it! |
| 459 | If you love it, there is no reason to not do it. |
| 460 | If you need to gain money, please search for it in another career. |
| 461 | If you really like science, just go for it. |
| 462 | If you really like the day-to-day of research, do not become a faculty member. Faculty members handle a lot of writing and administrative work. Being a full time research scientist if a better way to have a research career. |
| 463 | If you want a work-life balance and to be successful, then an academic research career may not be the right choice. |
| 464 | If you want more money - go to industry. You should be truly dedicated to the academy |
| 465 | If you would have asked me two years ago (when I was just two years into my postdoc), I think I was more positive about academic science. Unfortunately, academic science is not equitable and the funding situation is dire. At this rate, we will continue to see academic science as very white and male and 40% of the grants will continue to go to 10% of the labs, instead of be spread to more junior researchers (especially under-represented scientists). I think everyone who is in science at a junior stage should not be afraid to reach out to many different mentors and learn as soon as they can what options there are. It should no longer be the norm that we are in this to all become academic PIs, do what makes you happy and supports your goals! |
| 466 | If your goal is a faculty position: pick a PI with the funding to allow you to complete your projects. Complete your projects, publish, move on. If your goal is to do research: pick a PI with the funding to allow you to complete your projects. Have fun. |
| 467 | In my opinion if you are a WOMAN and have small children, and want to be a present and active parent, academia is not the way to go. Otherwise, I feel it can be a rewarding career if you respond well to stress and pressure, and can accept that these academic institutions are not part of the real world and have rules of their own. |
| 468 | In my opinion, it is absolutely important who your mentor is and how much they willing to invest in you and your career. As a postdoc, there is very little chance of you transitioning into an academic position unless you are from a lab that has money and reputation. PIs' spend a lot of time writing grants, leaving very little time for mentoring. In short, I would strongly advice against thinking of pursuing an academic career. |
| 469 | In order to achieve a successful career in academic research, one needs to understand early enough that it is more than a job in science - it a permanent dedication to scientific topics, with a lot of workload for a single person. Persistence and patience are essential traits to succeed. Networking from the very beginning is also of great importance to establish contacts with collaborators. Overall, research has become a multi-task endeavor. It's crucial to always stay up-to-date about new methods and technologies. |
| 470 | In this political climate and within the current academic system (funding, tenure, etc) an academic career seems more and more elusive. |
| 471 | insist on and work hard and come on |
| 472 | Interest is the most important. |
| 473 | Is it the most important thing you want to do in your life no matter what? |
| 474 | Is not for everyone. If you're passionate about science, research, and training, you believe you're good at writing and developing new projects, go for it. Please, don't consider this as the only respectable career for a scientist. Keep your options open. |
| 475 | It comes with a lot of challenges. There is a lot of self attained success in this field. |
| 476 | It doesn't come without hard work and learning from past mistakes |
| 477 | It is a great career choice, but you need to put the right effort. It is not a usual 9-5 job. |
| 478 | It is a great career. There is nothing in this world that gives you more satisfaction then transferring your knowledge to your students and next generation. |
| 479 | It is a lot of work, and would be difficult to balance work-life if you wanted to be very successful. Industry would pay better and not require you to sacrifice as much personal life. Make sure it is what you want to do before you pursue that route. |
| 480 | It is a path requiring a lot of sacrifice, a lot of work, and that necessitates a great passion and self-motivation. If you do not care or are not passionate enough about the science you do, the work-intensive postdoc years and the hassle of grant writing and permanent instability together with relatively low wages compared to an industry position may make it not worth the effort. Think about your priorities and your dreams; about how your work reflects to your personal and family life. For example, it is much harder to be a successful professor with 5 grants to your name if you have kids and want spend time with them; you will always be at a disadvantage compared to a person who doesn't have children. If you find that your passion and drive are strong and you want to give it a shot, do it - but be ready to work hard. Do not hope for a tenured-track position. There are good chances that you will be on soft money for the whole of your career. If your spouse works, him/her not being in academia is a plus (not dependent on government money and budget). If your spouse doesn't work, you may be able to have the family life you want and still work hard enough, but may ot see your kids grow up as well as you may want to... |
| 481 | It is a stimulating career, but reaching high levels is a complex path, driven by a combination of scientific and non-scientific factors. Faculty positions are extremely competitive and require a huge commitment and sacrifice |
| 482 | It is a very rewarding but challenging field. You have to love the science. It's isn't a job you can coast in. |
| 483 | It is absolutely vital to spend at least one year actively doing research in a lab before deciding to make it a career. I didn't have that experience before pursuing my PhD, and had no idea what I was getting into; I just wanted to increase my job prospects. When I had a hard time in grad school, I just assumed it was because grad school is hard. I didn't realize it was because this wasn't the career for me. I think if I'd had that experience earlier on, I would've been able to make a different choice. |
| 484 | It is an extremely tough path to take, and you absolutely should obtain hands on experience in a research lab before committing to it. You have to love it more than anything because the status of the field is gloomy to say the least. It is much more political than one may think. |
| 485 | It is difficult to provide advice considering I have not make it through an academic research career, and I am not sure of what to expect after a postdoc position. My provisional advice would be to look up career development programs at very early stages of your graduate school and/or postdoc to find out all the options available and that suit you the most. Also, always keep in mind that you and your mentor in academia have opposite interests (i.e. you want to leave the training position and your mentor needs these positions in his/her lab). This does not mean that the mentor/postdoc relationship will not work; it is just important to realize that there are biases when it comes to deciding your career plans. |
| 486 | It is high risk and stressful - have a good support system in place. |
| 487 | It is highly unpredictable and volatile and stressful. Do it only if you are sure that it is personally meaningful to you. |
| 488 | It is important to choose productive collaborators and mentors. |
| 489 | It is important to know that there are limitation of time, fund, position and idea. It should be well planned and guided. Also network with other colleagues and professors are important to improve all plan. |
| 490 | It is important to work hard, but more important to work smart! |
| 491 | It is labor intensive and one has to put in untiring effort always. |
| 492 | It is necessary to be passionate with your work, and really enjoy what you are doing to be successful and succeed in research field |
| 493 | It is not worth the pain. transition into industry or other non-academic paths after obtaining your PhD. If you do start a postdoc combine it with obtaining an MBA |
| 494 | It is probably not a very secure career path. Make sure that you are okay with the work outside the research, because as you get more senior that becomes a bigger part of the job. |
| 495 | It is something that you owe to yourself to try |
| 496 | It is very competitive and overcrowded. Unless they feel very strongly about doing research then I would dissuade them. It is not an easy career choice and comes with a lot of stress. |
| 497 | It is worthwhile to think about the factors that make an academic research career exciting and consider whether those opportunities exist only in academia or if there are multiple paths that will lead to an equally satisfying career. |
| 498 | It requires a lot of resilience! But opportunities vary a lot from place to place (country to country). |
| 499 | It seems to be hard and not always fair, but the reward is big. Make sure you have a really good idea of what a research career looks like in terms of work-life balance. |
| 500 | It takes a lot of time, effort and persistence, but is worth it if that's what you would like to pursue. |
| 501 | It will take longer than you think, and you will have to justify your choice to stay in academia every step of the way. |
| 502 | It won't be easy, its going to be a grind. Figure out early on if you can accept failure and still learn from it or be able to move on from it |
| 503 | It would really depend on the person. I have found out that this is not for me, but I can see how it would be great for someone else. |
| 504 | It's a competitive area with no guaranteed success, do it only if you love the science and big ideas keep you awake at night and not just for the laurels and admiration that comes from people with being an academic. If not, there are many other ways you can contribute your skills and brilliance to society and the world. |
| 505 | It's a constant process so make sure it's something you really see yourself doing for years to come |
| 506 | It's a great commitment. |
| 507 | It's a hard and long path. You will have a lot of discomforting moments. But if you are passionate by science and work hard you will be able to enjoy the few moments of pleasure of the career that make it worth. |
| 508 | It's a long and difficult path which requires a lot of persistence and determination, |
| 509 | It's a long process from the time you start your PhD. A lot of it depends on the lab you get in , the projects you handle and the kind of research contributions you will be able to make in a short period of time. Patience and Perseverance are the key. With decreasing grants and lessening of meaningful job opportunities, you should really thrive to succeed in the field |
| 510 | It's all about who you know. |
| 511 | It's all about your passion for the field. If you think you can contribute to the society and science, welcome! |
| 512 | It's hard and requires to much commitment and it doesn't always pays back |
| 513 | It's hard to continue along an academic career path for financial reasons. Relying totally on oneself is even harder; graduate student pay is low, and postdoc pay is hardly enough to live in a city and pay back student loans. I struggled. I would tell students to think about this before going down the path.. it can be difficult. But luckily there are programs like Loan Repayment from the NIH, which offer assistance post graduation. |
| 514 | it's hard work |
| 515 | It's not a meritocracy. Either you have to publish a lot, or publish in high impact journals only. In addition, you must be able to show that you can secure funding. |
| 516 | It's not an easy path and there aren't enough jobs |
| 517 | It's not as fun as it sounds. Go to industry. |
| 518 | it's not for everyone, you have to work hard, be dynamic and be lucky. you can spend long periods of time being frustrated with nothing working. you have to love the science and be passionate about your work. networking with peers and labs you're interested in and offering your help or services or forming collaborations provides vital long-lasting and fruitful connections. its often who you know that counts in terms of publications. get involved with excellent research labs, learn from them and become a vital part of the team. |
| 519 | It's not for everyone. |
| 520 | It's probably not worth it |
| 521 | It's really daunting so you had better really want it. |
| 522 | It's stressful and not always rewarding, but hopefully worth it in the end. If you are not fully committed you won't make it. Other careers have more perks and stability. I would choose this field if you find pleasure in constant challenge and you're able to take risks financially as it's not guaranteed that you can maintain your salary or position long-term. |
| 523 | It's very difficult, so don't keep your hopes high. Need to be ready with plan B. |
| 524 | It's very difficult, there's a great degree of luck involved, there is nepotism to be aware of, and don't pursue it unless you love research more than anything else. |
| 525 | It's very important to identify the 'Real Question' in research field. Only after that one can have a work plan to answer that question. one should know how to read between the lines and also how to correlate it with bigger picture. |
| 526 | It's very time consuming, it is tough. Given today's economic and funding situations, I would suggest using extreme caution as which lab to pick and which area to stick to should you decide on taking an academic research career. |
| 527 | Its a good area but i personally have more interest for Industrial Research Work. |
| 528 | Its a great job with a lot of freedom but its hard work, you need to be dedicated and to like being in charge of your experiments/time. |
| 529 | Its all about work satisfaction and nurturing your curiosities. |
| 530 | Its hard. There's not a lot of good guidance so you will need to teach yourself a lot of things. |
| 531 | Its really nice world to be. uncertainty do exist but the freedom do exist to move towards path not transverse by other. Life is beautiful but never to think about the monetary gains. Academic research career is really amazing path in life. |
| 532 | Its very difficult but do it not for sure self do it for the society and mankind betterment |
| 533 | Join a high profile lab and get a high impact publication, or, obtain your own funding! |
| 534 | Join a new professor's lab so you can see the process from the beginning. Join a small lab so that you will be able to help with lab management somewhat. |
| 535 | Join a PhD lab that gives good prospects in academia (e.g. graduated a number of students who obtained faculty positions). The lab culture and publication strategy is important. |
| 536 | Join the well renowned research group and start networking from the beginning. Good publications often comes from collaborating. |
| 537 | Just because you worked hard doesn't guarantee anything. However, if you keep at it, it's rewarding eventually |
| 538 | Just don't. |
| 539 | Keep being motivated |
| 540 | keep connections open-- never know when a job opportunity may arise. |
| 541 | Keep going no matter what you face |
| 542 | Keep going until they tell you no. |
| 543 | Keep good communications |
| 544 | keep on learning about new technologies |
| 545 | Keep reading broadly and going to seminars to stimulate yourself with new ideas. It's easy to turn off your brain and just keep grinding, but we often get stuck in a rut. Make time for deep thought about your project and broad thinking about your field and you'll develop ideas. |
| 546 | Keep try |
| 547 | keep trying, but have a good therapist. |
| 548 | Keep your eyes peeled and options open - there are fewer faculty positions than # of postdocs. Those really passionate about science should pursue this path and others should look around for something that fits them better. |
| 549 | Keep your options open |
| 550 | Keep your options open and explore multiple skills that can aid you in choosing the right career path. |
| 551 | Keep your options open for other career opportunities. |
| 552 | Keep your options open to other career paths as well. |
| 553 | Know that being a PI is about management, writing, and collaborating with others, not about conducting bench science. |
| 554 | Know the pros-cons, the pressure, alternative career choices; & finally take an honest self-evaluation to assess if this field is for you |
| 555 | Know what this career involves/requires. Not just during your many years of training, but also if (and this is a very low percentage of those that attempt this career path) you achieve a tenured faculty position in academic research. Ask yourself if that is the lifestyle that you would want to have, and if you have a partner or children, if you can fulfill your responsibilities to them during the entire course. |
| 556 | know what you sign up for |
| 557 | Know what you're getting yourself into and your limitations, and be curious about all career paths. |
| 558 | Know who you are and what is the most important thing in your life and keep that in mind. |
| 559 | know why you want it, know where you want to go, know people at the places where you want to go, choose department carefully, consider if negative financial impact of academic career is sufficiently satisfying tradeoff for academic freedom |
| 560 | Know why you're doing it, because the nights are long and full of terrors |
| 561 | Know your options |
| 562 | Know, explore, and consider all of your career options, including those in industry, those outside of science, and careers that include science but are off the bench. |
| 563 | Lack of funding, uncertainty, and lack of training provided in the academic labs should deter the most from the training for an academic research career. |
| 564 | Learn a lot from different fields and keep options open in realms other than academia. |
| 565 | Learn about all the other options for scientist careers too |
| 566 | Learn about all your career options first! |
| 567 | learn about the breadth of career paths and options available for doctorates |
| 568 | Learn how to critically read data and develop independent ideas and experiments. Work hard both at the bench and at understanding and staying current in the literature. Learn to ask for help and take criticism. Build your professional network to include scientist of various backgrounds and expertise. Meet and discuss your science as frequency as possible with these colleagues |
| 569 | Learn how to work efficiently and learn how to efficiently develop products (i.e., grants, publications). |
| 570 | learn how to write a grant as early as possible. |
| 571 | Learn to accept failure. |
| 572 | Learn to communicate well, about your science but also with peers, supervisors and mentees. Talk about your science and build a network for collaboration. |
| 573 | Learn to cope with failed experiments. Failed experiments do not equal a failed career. |
| 574 | Learn to structure your work yourself, there is zero structure given. Learn to find motivation without relying on any feedback. |
| 575 | Learn what skills you need and focus hard on those skills. |
| 576 | Like most careers it is challenging and it can be difficult especially to find and secure funding. I would not have chosen this career if I did not enjoy science and basic research. For me my career is my hobby. As with everything in life if you love it enough you will be ready to deal with all challenges. |
| 577 | Long haul, 10yrs+ of 60-70hr weeks :) |
| 578 | Look at your PI. Do you want to do what your PI is doing? |
| 579 | look for other options too |
| 580 | lot of work, and little money |
| 581 | Love it or get out. Work hard, work smart, network, think creatively, be passionate, mentor students and peers. But seriously, if you don't love it enough, don't take up the space of someone who really wants to be here. |
| 582 | Love your job |
| 583 | Maintain your frustration threshold as low as possible. Science is hard, don't despair too early. |
| 584 | make a lot of connections and collaborate as much as possible |
| 585 | Make a physical, poster style, week by week five year and ten year plan of where you want to be, and the targets you want to accomplish at each step. Include article deadlines, conferences to attend, abstract, grant submission and award deadlines. Then visit it every few months to see where you are. |
| 586 | Make sure it is really what you want, because the preparation for a research career is different from a teaching or non-academic career. |
| 587 | Make sure it is what you are extremely passionate about. |
| 588 | Make sure it's something you love, because the its a hard road, especially if you don't. |
| 589 | Make sure it's what you want to do. |
| 590 | make sure that you really want it |
| 591 | Make sure the lab has a record of publishing in big journals and make sure your PI has a specific project lined up for you. Ask the people in the lab if they trust the PI's ideas for projects and whether they produce publications. |
| 592 | Make sure there are back up plans |
| 593 | Make sure to choice your mentors carefully. A good mentor will support your ideas and goals. |
| 594 | Make sure to choose a lab that publishes regularly and a mentor who will actually act as a mentor and not just get science out of you. |
| 595 | Make sure to know what your other options are/have a backup plan. I should follow my own advice. |
| 596 | Make sure to not go where everyone else goes in your field of research (competition is very high there). Be aware that the salary is comparably low and the work load is extremely high. Your spouse/kids have to be prepared for that. The transition from lab bench to desktop is sometimes brutal and often boring. Learn early to speak to local press and donors about science since NIH and NSF grants become increasingly difficult to obtain. Above all: Love what you do and do what you love! |
| 597 | Make sure you are a hard-working, persistent, almost ruthless, and patient person. |
| 598 | Make sure you are aware of all the sacrifices and stress that this career will create. |
| 599 | Make sure you are aware of the challenges before beginning |
| 600 | Make sure you are passionate about what you do. |
| 601 | Make sure you are ready to leave the bench |
| 602 | Make sure you care deeply about research before you commit your life to it, and have a backup plan in case you have trouble securing funding. |
| 603 | make sure you find a good mentor, be persistent |
| 604 | Make sure you follow your passion |
| 605 | Make sure you have strong writing skills and a high ego to sell yourself and your research. There are a lot of politics that goes on in the research world and you need to be prepared for bruised egos and petty squabbles. |
| 606 | Make sure you know what type of job you want before enrolling in an educational program, because most PhDs end up not working in academia and would not necessarily need the PhD for jobs they end up taking. |
| 607 | Make sure you know what you are getting into (e.g. failed experiments, rejected grants, poor job prospects) and that your love of the work will be enough to bring you through these hard times. |
| 608 | Make sure you know what you are getting yourself into. Make sure that you know what kind of options will be available to you 10 years down the road. Be prepared to make sacrifices and deal with uncertainty. |
| 609 | Make sure you know what you're getting into, research your supervisors well |
| 610 | Make sure you know why you want this career because the road is not easy and more work does not always guarantee you will achieve your goals. |
| 611 | make sure you love doing research |
| 612 | Make sure you really care about answering specific scientific questions rather than caring about success. It will keep you motivated through career slumps. |
| 613 | Make sure you really enjoy coming up with your own hypotheses, have the knowledge to assess their novelty, and the writing skills to get them funded. Also, because it is so competitive, I don't think it would be a fun position for someone who didn't really enjoy/feed off of criticism (constructive or otherwise). |
| 614 | Make sure you really want it and that you have back up options (e.g., clinical training) in case you cannot secure funding. |
| 615 | Make sure you really want it. |
| 616 | Make sure you talk to a lot of different people. Although you have freedom to research your own ideas, the funding climate now is not great and it is becoming extremely more difficult to get funding for your research, especially if it is not in a 'hot topic' area. |
| 617 | Make sure you truly love the job and have a strong passion for it before choosing this path. I thought I did, but years of doing this work burnt me out. Even if you know you could be successful, think about if this is the lifestyle you really want. Also, think about all of your career options early in graduate school. |
| 618 | Make sure you understand the amount of time, effort, and commitment required to reach your specific goals in research. Seek mentorship early in your career and get help from those who have successful careers. |
| 619 | Make sure you understand what is involved and how competitive and/or difficult it can be to get funding in certain field or areas of study. |
| 620 | Make sure your heart is 100% in it. If not, you will not be successful. |
| 621 | Meet in person and get to know well your supervisor before accepting the job offer, because sometimes they show their true colors later and you might not like them! |
| 622 | Mentor/teach throughout, learn to write grants, focus on research, but not solely on research |
| 623 | Mentors are very important. More than one ideally... Talk to as many people in your field as possible. |
| 624 | Mentorship is key. |
| 625 | Multi-tasking is an extremely required skill. Ability to generate high quality data for publication in journals, while simultaneously mentoring students, writing grants and manuscripts, as well as reviewing submissions by others. |
| 626 | My main advice would be DON'T. It's very long and tough journey. I am 35 and I am not making enough money to support myself alone, I cannot imagine supporting a family. |
| 627 | My main motivation for pursuing this career path is that I want to direct my research and contribute new knowledge to my field. I think if one's motivation is glory or something similar, s/he is likely to be disappointed. |
| 628 | n.a |
| 629 | N/A |
| 630 | Need to be willing to work very hard for low salary. Be ready to sacrify your personal life. Be patient and persevering. If you are really passionate and want to feel useful for others it is a good choice, if your are looking for money and fame, do something else. |
| 631 | Need to network and find a supportive environment |
| 632 | Network and get to know as many people as possible who are doing the job you desire. Ask them about what it takes to be successful as independent investigator, what are the challenges, and what are the rewards. |
| 633 | Network well. Have a plan B (and C and D) because hard work is not enough - you need to get lucky. Everyone who succeeds works hard, the inverse is not true. |
| 634 | Never choose academics...never desire to b a postdoc |
| 635 | Never forget that the goal of research biomedical research is to eventually find targets for human health issues that will hopefully help eradicate disease... and that research does not occur in a vacuum and requires passion, leadership, hard-work, and collaboration. |
| 636 | No matter how good you are you need very good connections and networking and you need a large amount of luck and being in the right place at the right time. |
| 637 | No money, no security, no vacation, no guarantees. Your passion for science has to be the only driver. |
| 638 | No pain, no gain |
| 639 | none |
| 640 | Not enough grant funding available |
| 641 | Not everything is a high-impact publication |
| 642 | Not sure |
| 643 | Not the right time. Everyone doing science doesn't get a Nobel prize. Science is an old scientist's club hanging out scratching each other's back and staying silent on your friends mistakes, when a postdoc or student complains. Also, if u have been a scientist for 20-30 yrs, ur ideas will succeed, ur postdoc will succeed, and your student will get all the prizes and possibly grants..it's a biased system now. Therefore, not the right time..let FOR change it and then jump in ;-) |
| 644 | Not worth it unless you are passionate about research or the cultural environment is essential for your happiness. |
| 645 | nothing |
| 646 | Obtaining and maintaining an academic research career requires a lot of passion and idealism about the scientific process. |
| 647 | One should pursue academic research career based on their interests and passion. It requires lot of commitment, so if you have to be ready for that. |
| 648 | Only do it if it's what you really wish to do |
| 649 | Only do it if you absolutely love the work. |
| 650 | Only do it if you can't imagine doing something else |
| 651 | Only do it if you go into it having an idea of why you are doing it and what you want to a PhD for. Go to big name labs. |
| 652 | only do it if you have good reasons for not wanting to do anything else and if you can live with the uncertainty / starting over in a different career after you fail doesn't bother you |
| 653 | Only do it if you have questions you deeply want to know the answers to, and don't mind playing a bit of 'the game.' But also know, as you and your peers become frustrated with aspects of the career, that other career choices include similar challenges. Think of an academic career list being an entrepreneur and starting a company- it's grueling and will take years and you constantly have to think about funding and being unique and getting there first, but if things get off the ground and you see YOUR ideas come to life and spread and influence others, it's very exciting. |
| 654 | Only do it if you know it is what you want to do. There are too many unreasonable people trying to build their reputation on your work. The fields are all becoming more competitive. There is less research funding. Their is no job security, even with tenure. Also, their is not much salary. |
| 655 | Only do it if you think of it a hobby more than a job |
| 656 | Only do it if you're really passionate about it and can deal with constant failure. |
| 657 | Only get in an academic career if you can deal with disappointment and failure everyday. |
| 658 | Only if you thoroughly investigate and understand how many other options there are. If academic research is still your top choice, then go for it. Most of the time postdocs are blind to career options and don't take initiative to explore, so they remain on a less than ideal career track. |
| 659 | Only pursue an academic research career if you cannot picture yourself doing anything else. It is not a safe bet if you are interested in making money, or having any personal time to pursue non-research interests or spend significant time with family. |
| 660 | only pursue if you are passionate about research |
| 661 | Only pursue it if you are truly passionate about research. If not, you will be extremely unhappy and stressed. Research is a career that does not pay as high as others for the amount of time you put in (e.g., education, postdoctoral fellowship, hours at the office, hours at home, hours even during vacation). It never ends. |
| 662 | Only pursue it if you're 100% certain this is what you want to do. And always have backup options, the amount of positions available are already low and may decrease with potential budget cuts |
| 663 | Only pursue it in extremely successful labs (Nobel laureates, HHMI, etc), and only do it if you are sure there is nothing else in the entire world you would even consider. It is brutally competitive. |
| 664 | Only pursue this path if you can not find similar satisfaction in a well-paid and/or non-research job. |
| 665 | passion and quiet |
| 666 | Passion for science and discovery. Ability to acquire grant and publication in top journals |
| 667 | Passion for science is a must. Endurance, perseverance, great grant writing skills. |
| 668 | Passion, persistence and dedication |
| 669 | Patience and Persistence are important characters to pursue a successful academic research career. |
| 670 | Patience!! |
| 671 | Pay close attention to the department and higher up co workers. Politics will play more of a game for you then I was comfortable with. |
| 672 | Pay close attention to the job market outside of academia and try to develop skills that will allow you to transition out of academia as early as possible. Post doc position seems to be a dead end job if you don't want to stay in academia. |
| 673 | Perseverance |
| 674 | Perseverance and hard work is the key. |
| 675 | Persevere, and try not to let the politics of science get you down. I think particularly for postdocs who are minorities in some way (gender, race/ethnicity, sexual orientation, etc.) the entire culture of science can feel adversarial. But the more of us who stay in the field, the more the climate changes. I've only been working in research for ten years and things have already changed for the better. |
| 676 | Persevere! It's subjective and involves constant rejection. You have to truly love it more than anything else. |
| 677 | Persistence is key. |
| 678 | Persistence is one of the keys to success. |
| 679 | Pick an exciting research topic that can lead to publications in top level journals and let you secure grant funding |
| 680 | Pick the researcher with the recent string of top tier journal publications, and try to work with them. Work very hard to make those publications. Do not fear getting help from outside of your laboratory to supplement mentorship you may not receive from your mentor. |
| 681 | Plan ahead and add to your resume each year. Seek out opportunities for grant writing, presenting, and mentors. |
| 682 | Plan research initiatives 2-3 years out |
| 683 | Plan your career ahead It's hard to make a good choice for your lab when you are a graduated student because of the lack of experience, however the choice of lab for a postdoc is crucial and should be made carefully with a lot of consideration for your career objectives |
| 684 | Plan your steps carefully. A poorly chosen mentor can have a strongly negative impact on your future. |
| 685 | PLAN. Plan as soon as possible, how are you going to get there? Find mentors, GOOD ONES, people that will support you, encourage you and critique you if needed. Go outside your comfort zone of the lab, engage in science outreach, network, interact! Science is more than working hard at the bench, and I wish somebody had told me that earlier in my career. |
| 686 | Please change your thinking |
| 687 | Please choose this only if passion/intellectual quest has driven you here. If you are here for ANYTHING else you are likely to be disappointed and fail. |
| 688 | Please consider other options in the process and keep an open mind toward other careers! Best of luck! |
| 689 | please look for something else first |
| 690 | Please make sure you understand the amount of sacrifice and commitment required to be a full time researcher. |
| 691 | Please think of what you would want in the future before starting your career |
| 692 | Politics counts |
| 693 | Potentially reconsider. It is getting tougher and tougher. One should only do this if one cannot imagine doing anything else. This has been true for me since I was 16 yrs, but these last 2 years of postdoc have finally made me think about other career options - despite the fact that I am 'successful'. I don't want my kids to go into research; I want them to do something where their work and knowledge is actually appreciated. And where this appreciation is reflected in the salary. |
| 694 | Practice networking. We like to think that academia is merit based and it's not. Who you know and what they think of you is more important than the number of hours that you work. |
| 695 | Practice resiliency. That is the most valuable trait in the field. Talent and smarts is not enough- must be doggedly determined. |
| 696 | Practice writing |
| 697 | Practice writing grants, talk to new PIs, and get a sense of the amount of work and dedication it takes to be successful as an academic researcher. Also be realistic about grant availability and how long it can take to become established. |
| 698 | Prepare a plan B. |
| 699 | prepare to be poor. have a backup plan. |
| 700 | Probably don't |
| 701 | Produce quality science |
| 702 | Publication in high rank journals |
| 703 | Publish and network |
| 704 | publish and try to get funding, have novel ideas |
| 705 | Publish in high end journals or chose another career |
| 706 | Pursue a computer science degree or skill set as early as possible. Not a wise decision to pursue life science career considering the efforts that will be required. |
| 707 | Pursue a field you are passionate about. |
| 708 | Pursue an academic career based on how much you enjoy science in spite of the potential for many failures. If you don't enjoy the process, do not pursue an academic research career. |
| 709 | Pursue an academic research career as long as your passionate about the path and are willing to put the effort. Keep on mind that there are alternative career paths equally fulfilling, so there is no frustration in case you have to switch paths. |
| 710 | Pursue only if you are absolutely sure about it; and if when there you see it's not for you, leave as soon as possible, as the longer you wait, the more difficult for you to leave it will get. |
| 711 | Pursue this career if you have a strong passion for research. |
| 712 | Pursue your passion, do not be discouraged by setbacks, and find a healthy work-life balance. |
| 713 | Pursuing an academic career in Biomedical Sciences means high risk, many years of low-income, high likelihood of unhealthy life style, and lack of time to accompany your family. Be cautious before you make the decision to sacrifice yourself and your family. |
| 714 | Pursuing science for the sake of exploring how the world around us works, and planning an academic research career are two very different endeavors, which require many mutually exclusive skills. Working towards a career in science must include careful choice of mentors, labs and projects in graduate school and post-doc. Papers are the currency of research, so it is crucial to publish extensively in reputed journals. In the job market, being able to market your 'brand' of science - topics, approach, methods, etc becomes an important ingredient to success. Having said that, collegiality remains a desirable quality of a successful job applicant in academia. |
| 715 | really be passionate about it, consider alternatives and pursue steps towards them early on, seek mental health help when needed early on |
| 716 | Really make sure that this is the path for you before pursing a postdoc. Make sure that your spouse/significant other understands the time commitment that you have at your job and that will be fully supportive of you and your career. |
| 717 | Really think about your career choices, both immediately and your long-term plans. An academic research career should not be your default decision after graduate school. It is great if you find something you are passionate about and have some luck in your experiments, but it has little security, and effort counts less than results. |
| 718 | Reconceder, it does not worth. |
| 719 | Reconsider. The competition for faculty positions and grants is incredibly fierce and success comes down to luck as much as credentials. |
| 720 | requires a long time, dedication, and hard work. Need to look for a lab with a good track record and sufficient funding. Need supportive mentors. |
| 721 | Research can be both a highly challenging and highly rewarding field if you are working on something you truly find interesting. It is worth keeping in mind that you are competing for limited resources with colleagues that are as smart/hardworking as you, if not more so. There are few other career paths that you have to constantly compete in order to simply continue to work in your field. This is something to keep in mind as you pursue a career in research. |
| 722 | research career is not as common job, its time demanding and high degree of commitment |
| 723 | research career options early and often. reassess constantly. do what makes you personally happy even if it does not come with the largest fiscal reward. |
| 724 | Research career tracks (e.g., professional lifestyle, compensation), introspect to discern lifestyle wants and needs, and deliberate with loved ones and trusted colleagues. |
| 725 | Research doesn't make money. You are a scientist because you love science and knowledge |
| 726 | Research is long-lasting work,we need more patience.Work hard,work smart, don't rush to expect too much at your beginning! reach our goals step by step! |
| 727 | Research is not just a job it's a lifestyle think about it. |
| 728 | Research should be a passion not one of many job opportunities. |
| 729 | Research the field before joining. |
| 730 | Researcher daily tasks rely on numerous skills like writing communication, teamwork, problem solving, planning, learning, self-criticism, etc. I think that people wanting to pursue an academic research career are mostly driven by a love for some aspect of the scientific process, like problem solving or writing. It's reinforced by the relative freedom that we have compare to other jobs. This mean that during our training we have a tendency to neglect or avoid working on other necessary skills. Thus my advice is simply to force yourself to work on unpleasant tasks. |
| 731 | run |
| 732 | Run away, it is completely not worth it! You will be mentally run-down, under-paid, under-appreciated, and in the end make less than you're worth with fewer benefits. |
| 733 | RUN! |
| 734 | scary funding climate |
| 735 | Science is somewhere between art and engineering, both in terms of pay and expectations: you don't get paid a lot, you must always be creative, there is an element of luck, and you have to have discipline and a focused mind. It is psychologically challenging but fulfilling. |
| 736 | science should be your number one passion. It must be strong enough to overlook the many long hours and the fact that you're spending your peak earning potential years in a stressful, low-pay, unstable 'training' position. |
| 737 | see statistics and be informed |
| 738 | Seek guidance and perspective from several sources first. |
| 739 | Select the best university you can, and then the best PI/Professor/Mentor/Supervisor available before pursuing a PhD degree. The best training/study/project/thesis makes a really huge difference. |
| 740 | Self-motivation is absolutely required. |
| 741 | Seriously rethink your career choice. While the work is great and fulfilling, the funding is sparse, positions are sparse and it is not a clear cut defined career path. |
| 742 | Show us more details and plan for the career |
| 743 | Some of success in academia is luck, you can't always predict which project will give you the best results. Be open to unexpected findings, they have the potential to be useful and interesting lines of research. |
| 744 | Some of the key characteristics to succeed I think is resilience and ability to focus constantly toward finding a solution to a problem. Another vital aspect would be to take small steps towards the bigger goal and being happy when the smaller goals are achieved. |
| 745 | Start early |
| 746 | Start early and work hard to publish frequently and in top quality journals. |
| 747 | Start early in graduate school to know what are needed in your CV and what are the timelines you need to watch for. Be focused on publishing papers which is even more important than learning skills. Read broadly! I made the mistake of being very technically skilled but not reading broadly, stayed in PhD lab as a postdoc for too long after defense, and was too nice to say no and push my papers out as fast as I could have. |
| 748 | start early, be aware of the impact of various early research choices on the chances for success in the futuer. i.e. utilizing novel methodologies, learning something praised in the field, having a successful PI are very important and may be 'worth' more than going to ones filed of interest at an early stage. |
| 749 | Start getting involved with research as early as possible, do everything you can to contribute to manuscripts, and don't be intimidated by the publication or grant funding process. Try to apply to grants and publish papers as soon as you can, and you will improve at it by the time you are a Postdoc. |
| 750 | Start learning what the career entails early, don't only focus on the science. Keep an open mind - learning about other career opportunities will help you be a better mentor as a PI should you stay in the academic research track. In your career, you are bound to have at least one mentee who is interested in a career that has a different trajectory than yours. Do not dissuade them, encourage them by being knowledgeable. |
| 751 | Start looking into non-governmental funding options in your field. |
| 752 | Start one only if you are truly passionate and driven about science as frustration is constantly present. |
| 753 | Start your training in a well-known research lab. Your scientific pedigree is very important. Also, don't do it if you want to have a good work-life balance. |
| 754 | Stay focused and be persistent when things donot work as expected. Find a good circle of mentors and be good at networking and collaboration. |
| 755 | Stay focused, publish... publish better |
| 756 | stay foolish, stay hungary |
| 757 | Stay tough and positive |
| 758 | Strive hard but do not expect equivalent rewards. |
| 759 | Successful academic career requires a sacrifice. If you have other priorities in life, such as life-work balance, give it up. I had that motivation in the past, but these days, having a family, has changed my perspective. I intend to look for a less intensive scientific career, after this postdoc. The second advice is that you must have the passion and curiosity, otherwise it will tear you down, because these traits allow you to overcome frustration and recurrent failures in research. |
| 760 | Successful academic research careers are hard to achieve. If this is the path you want to pursue make sure it is really what you want. |
| 761 | Surround yourself with good mentors and work harder than the next person. |
| 762 | Take into account , that not everything what you are plan and wish for is only dependent on you, there is a bug part that need luck. |
| 763 | Take ownership of your time and your work. Direct your efforts to the tasks that will benefit your career plans the most. |
| 764 | Take stock in your life and career goals and understand yourself. |
| 765 | Take the time to explore your options and choose research only for yourself, ignoring the outside influence of family and friends. |
| 766 | Take the time to talk with principle investigators of various experience running their laboratories -- well established PIs, recently stabilized PIs (e.g. those that have just acquired multiple R01 grants), newly minted PIs. It is really worth considering their perspective on what an academic career entails: grant writing, peer review responsibilities, how to publish papers, what research topics can and should be considered, what research topics might be unfeasible, how much direct and demonstrable research experience you need to start a laboratory in a certain topic, how much creativity you are 'allowed' to exercise in selecting projects, the concept of passions vs. reality. Overall I think a very very clear map of the benefits and drawbacks needs to be established. |
| 767 | Take time before grad school! |
| 768 | Takes enrmo amounts of patience, hard work and tenacity with some amount of luck. Be at the right place in right time |
| 769 | talk to as many people as you can who are in the field and ask very thoughtful and detailed questions |
| 770 | Talk to as many people as you can, from all different 'kinds' of academic research. There are lots of ways to be successful. |
| 771 | Talk to everyone who will listen and answer questions, from graduate students to senior faculty, and don't stop, even after you make a decision. Everyone has different perspectives, and only by taking in all of these opinions and evaluating their relevance and value to you can you truly understand your own goals and whether they will be satisfied by your career choice. |
| 772 | talk to faculty at many different institutions. talk to those who decided not to go into academic research. Work through the book what color is m parachute. Think about how you like to work best..in groups, independently, formulating your owns ideas, taking directions/plans from others and putting them into practice. |
| 773 | Talk to other people in the position, get good publications, apply for grant funding |
| 774 | Talk with many people |
| 775 | Talk with someone in the field, specifically to young investigators or senior postdocs. They are the ones who know what's the rewarding and frustrating sides of academic research. |
| 776 | Talking with other scientists is very helpful. Try to fight off the idea that others are trying to steal your ideas. Science works best when we freely share ideas, experimental pitfalls, and reagents. |
| 777 | That environment matters. Being at a supportive institution with excellent mentorship and opportunities in your field of choice will be important for success. |
| 778 | That it is a mafia and one needs to sell oneself properly before being able to do 'any' science. There is more of bragging one needs to do before implementing science. If you are passionate about science, that alone is not sufficient. |
| 779 | That it's incredibly competitive and not very likely that you will be successful, especially given the funding situation in our country. Leaving and working outside the USA may be a more viable option for many. |
| 780 | That it's probably a bad idea. |
| 781 | The best insight I have into what different research careers might look like are from seeing my friends and colleagues take various paths and hearing about their successes and struggles. |
| 782 | The chances are: you are very likely to fail and end up with a postdoc position (or something similar) for the rest of your life. |
| 783 | The chances of gaining a tenure-track position are abysmal and the chances of remaining funded throughout your career is even worse. Consider a career in industry, or obtaining funding through sources other than federal research grants. |
| 784 | The choice of the PI for a postdoc or even as a PhD student is critical. The philosophy of the PI should be to reward your efforts by publishing quickly your results because if you don't publish as postdoc your academic career might be jeopardize. Also, I think it's important to have managerial training even for academic career. |
| 785 | The climate it terrible. The NIH is attempting to help with grants, but the researchers on study sections are still the ones giving them scores, so it isnt yet moving in that direction to help early career investigators. You need funding to get a faculty posistion, and you need a faculty posistion to apply for an R grant, so it is a bit of a conundrum, as faculty posisitions are dimishing, or hiring only well funded people. I am worried that at the emd of this year and this job search- I will be scrapping new career ideas together to be able to earn a paycheck. |
| 786 | The current mood of the people I know in academia (mainly other postdocs) is that post doc positions are dead end jobs unless you know you want to be in academia and have a chance of staying in it (extremely good publications, successfully written grants). I guess that's not really advice - my advice then is that you have to know you want that kind of lifestyle and be mindful that if you haven't checked the appropriate boxes (publications, grants) then it is extremely unlikely you will be successful. |
| 787 | The effort and the time that you spend planning your experiment and standardizing the readout is gold.. Don't try to guess, that is going to give you strong data |
| 788 | The first question is: do you like research? Do you like thinking up new research? Do you like discussing about research of yourself and others? Do you like selling your potential research, and believe in the power of you ideas? The second question is: do you like writing? Because it turns out that you must be a effective and productive writer to be a successful academic researcher. If the answer to these questions isn't yes, why not consider other options? |
| 789 | The new trend is personalized medicines and translation medical science. In order to keep a sufficient flow of grant money, one needs to understand not just the basic science, but also focus on the 'so what' question. One needs to highlight the impact of the research from a more translational aspect. |
| 790 | The only reason to pursue an academic research career is because you absolutely love the work that you do and highly value day-to-day satisfaction with your job. There are almost no other benefits of this career path! |
| 791 | The pursuit is worse than the work |
| 792 | The science is hard, but if you enjoy doing it - then keep on doing it! But remember there are many other things to juggle in academia than the science - Such as working with difficult collaborators or people with personalities which do no resonate with your own. Dealing with competition in academia among peers is difficult as well. The impostor syndrome can be extremely difficult to deal with if you end up having it. Don't let your self value end up only being measured on your research (easy to do for many passionate scientists). Follow what you enjoy doing, if you enjoy what you are doing and are passionate about it - there is a far greater chance of doing your best career wise and enjoying life. |
| 793 | There are lots of opportunities for becoming a faculty member at a teaching-intensive university. If you want to work at a research-intensive university, sell your soul to the Devil and be prepared to turn your back on friends, family, and everything else you might love in life in order to achieve what you must to gain that research-intensive position---unless you're an ethnic minority, in which case, people will throw grant money at you left and right and you can just coast on hard work (you won't have to sacrifice as much as your peers). |
| 794 | There are no relevant or worth while incentives apart from the pursuit of scientific knowledge, and for most people this is not and should not be enough. |
| 795 | There are pros and cons to a career in research. While the day to day schedule can be very flexible, there are times when you will need to work really long and hard days. While ~90% of experiments usually do not result in publication-quality data, the experiments that finally do are incredibly exciting/rewarding. Although progress can be slow at times, seeing the ripple effect of your work can be incredibly satisfying/exciting/motivating. If you feel comfortable managing those types of pressures/stresses, then go for it! |
| 796 | There has to be some personal attachment, investment, or whatever you want to call it; because otherwise when you look to all the careers it will be hard to fight the temptation. |
| 797 | There is a shortage of permanent positions. Therefore consider alternatives. |
| 798 | There is always someone smarter out there, but you can control how hard you work. Research, like many things in life, is a battle of attrition. Work hard in the lab, write a lot....like more than you think you should, read often, collaborate with others, keep growing. And most importantly, don't let a paper or grant rejection define you. Your worth is derived from the things in your control, not the things out of your control. Oh, and remember to have fun, because if you live to work, you will wake up frustrated and unhappy at 50 years old. Work to live. Travel. Live. Laugh. And don't take life too seriously, because none of us will get out alive. |
| 799 | There is so much I could say to answer this question, but this question is not asking for an essay for an answer. I would advise anyone that is considering pursuing a career in academic research or any other path to ask themselves why they are pursuing that particular career path. You really need to passionate about your craft if you want to be successful in life. Making a career in academia or many other fields really takes a good combination of intelligence, endurance, management, and selflessness to be successful. If you're not ready to do what it takes to become have a successful academic research career then you should pursue another career path. |
| 800 | They ned to be focused on their future career , what they want to become either wants to go into industry or staying in the research. Basing on their decision they can put their efforts. |
| 801 | They should do some internships to make sure they really know what is about and to know what they want. |
| 802 | Think a lot about your personal values and priorities and make sure they fit with realities of an academic research career. |
| 803 | think about alternate careers , weigh out pros and cons |
| 804 | Think about effort, compensation, and work-life balance. |
| 805 | Think about how much failure you can handle, if you have a low threshold, then it might not be the career for you. |
| 806 | Think about if it is truly what you want. I would also warn them about how competitive it is to succeed, even if you have done everything 'right.' It is probably important to make sure you are in a 'good' lab with a PI that is well respected in your field, but also one who is a good mentor. |
| 807 | Think about it again. If one does not LOVE it, do not do it. |
| 808 | Think about the entire landscape of the position. Academic positions, more than nearly any other job, require diverse skills and activities, many of which are counted upon by others. Be careful about moving forward if you're only interested in certain aspects of the job. There are much more accessible, rewarding careers that would undoubtedly suit you better and would better serve those who depend on faculty. |
| 809 | Think about the long training years and the low salary that requires to sacrifice other aspects of your life |
| 810 | Think about the work-life balance you want and which aspirations do you have in terms of economy for your future, and how you want to enjoy life with your family and friends. |
| 811 | Think about what you most enjoy spending time on daily, and then think about whether you would be doing that in academia. |
| 812 | Think about what your long term career goals are and what fields will be able to support academic researchers. |
| 813 | Think about why you want to do it and if you are convinced this is your thing, go try. |
| 814 | Think about work-life balance and the amount of time and effort required to gain an academic faculty position. |
| 815 | Think about your long-term goals and if it is something that you really want, don't let anything or anyone persuade you otherwise. |
| 816 | Think about your priorities. What you want to accomplish in your life and what's important for you. Talk to other people that made this choice - and talk to people at different stages of their careers. |
| 817 | think again and be very committed and goal oriented |
| 818 | Think again. The reality is that it will not happen. Try for you dream but have a strong backup plan that you also actively pursue. You can still make meaningful contributions to society without a faculty research position. |
| 819 | Think again. There is no future there. |
| 820 | Think again. There have to be better options. |
| 821 | Think ahead to what your CV must look like in order to obtain the job that you want and strive to make that happen |
| 822 | Think based on your capacity of enduring problems that could be regularly occurred in research field. It is not good at all simply sticking to the question if the research activity is interesting. |
| 823 | Think before you leap. |
| 824 | Think carefully |
| 825 | Think carefully about long term goals as well. Also think carefully about the time and effort it takes to have a stable research career while knowing that thew likelihood is low. It also requires careful self-evaluation to asses whether your CV is sufficient to obtain such a position. |
| 826 | Think carefully about whether you'll be able to find employment after you've trained for so long. |
| 827 | Think carefully about why you want this type of career, and what you're truly willing to give up to achieve this career goal. |
| 828 | Think carefully about your career trajectory EARLY. Think about grants and publications not just the research you're performing. |
| 829 | think carefully before starting a post-doc, if that is what you want to do. make sure to look at industry options as you are finishing your phd |
| 830 | Think hard about what the process entails and where you see yourself in the future and if this is the right goal for you. |
| 831 | Think hard about what your priorities are and why you are interested in pursuing a PI position. |
| 832 | Think hard, work hard, stay calm |
| 833 | Think it through. It's important to know what you really want and what makes you happy. I'm driven by my thurst for knowledge but what would be your intentions leading to an academic career? If its only the reputation you should look for other opportunities... |
| 834 | Think long and hard about what you want in the long run. Assess yourself, e.g. how well do you cope with stress and failure? Be honest about your expectations, and define your version of success. |
| 835 | Think long and hard about whether it's worth it, as it likely isn't. First, the bureaucratic inefficiencies bog you down and detract you from the actual fun of science. Second, after a certain point, your life is more about PR and management than it is about science. Third, the academic institutions arbitrarily devalue your contributions both financially and through the game of non-promotion. Finally, the most disheartening part of the game is the high degree of fickle whimsy and politics that scientists allow to bias grant and paper review/acceptance, such that the system rewards high-volume garbage/fluff producers and p-hackers to an inordinate degree, ultimately hurting the field. |
| 836 | Think long and hard before finalizing that decision - speak to people in those positions and get advice from them |
| 837 | Think more about the daily lifestyle that comes with research positions. Passion for science is only one (minor) part of this job. Are you ready for a semi-nomadic life, variable income, potential long hours and psychological pressure of uncertainty and rejection (experiments, grants, etc...)? |
| 838 | Think more than twice. Consider what really wants for his/her future. If have family, consider seriously the academic research career: if you value your family, time with your spouse and kids and enjoy the simple but blessed moments that only family can provide, I suggest follow another path, not research. |
| 839 | Think of alternate career, if you did not get a grant in the first 3-4 years. |
| 840 | Think of the prospects from a realistic standpoint and make sure to fully consider other options. |
| 841 | Think real hard about whether this is what you want to do considering you will continually fight to pay for your own paycheck. |
| 842 | think really hard about how this training will affect your future personal life both financially, emotionally and physically |
| 843 | Think thoroughly before you join the academic field |
| 844 | Think twice |
| 845 | think twice |
| 846 | Think twice and hold your luck close. |
| 847 | Think twice, but don't give up. Also there are many other options other than to be a PI. And to do a PhD and postdoc, especially in a different country is still very valuable experience. Hopefully useful for other jobs as well |
| 848 | Think twice, get prepared for an alternative way |
| 849 | Think twice! |
| 850 | Think twice. If you want stress free life with more money and work life balance choose something else, there are many career options. You will be successful if you will put this much time,efforts and brain in any field. But as i m doing it ...it just depends on interest and choices. |
| 851 | Think twice. It is a highly demanding lifestyle only suitable for people who value science more than other activities in life. |
| 852 | Think very carefully about what you want. Funding is poor, hours are long, and salaries are low. You really have to love what you do. |
| 853 | Think very carefully what you are getting into, and have a back up plan |
| 854 | Think very well which lab you are joining for your postdoc. Ask the people that are working there before entering. You need a place with good environment, creative and resourceful. |
| 855 | This kind of job requires a lot of patience, it is for the long distance runners. The rewards don't come so often, and there are a lot of disappointments along the way. |
| 856 | This career fits people that are achieving a lot from the beginning. The standing point is important. |
| 857 | This career requires hell lot of patience and continued effort and sincerity. There is continued struggle in one form or another. Immediate struggle is your Results, experimental failures. Intermediate struggles are Funding, publication and continued struggle is Faculty position, then grants, then Publishing as a PI and able to make a significant contribution. |
| 858 | This is a career path for those that wouldn't be happy doing anything else. If you know that, you have the motivation to succeed. |
| 859 | This is a conflict of interest, so I dont think it is really to my advantage, but since I have to enter to be eligible for the gift card I will say: The competition is fierce, so if you cant handle the heat or dont like the heat, get out of the kitchen. |
| 860 | This is a very broad question. But I have been asked for advice in this area ---mostly by undergraduate students ---of how to get into graduate school because they believe that they would like a career in academia someday. My advice to them is to get as much experience in research as possible. Also to get different types of experiences so you know what you like and what you don't like. It is critical that you are able to narrow your research interests down and that you can take a direction in your career. Also, it is good to have a diverse range of experiences that you can bring to the table. It has been important to me to have several mentors and to know when to seek out my mentors for support. Find someone's resume or CV that you admire and then identify what is missing on yours and work hard to improve the areas you need to be more competitive (e.g., publications, reviews, presentations). I have recently been offered a faculty position and have been very fortunate, but have worked really hard and taken advantage of many opportunities (grants, papers, projects, etc.). I have had some of the most amazing mentors and collaborators---find them, you will not succeed without them. This is critical if you want to work in research. |
| 861 | This is not an easy field. Tenacity is of utmost importance |
| 862 | This is such a tough choice. I think it's certainly a rewarding career path given that you can follow your own curiosity and questions. You may need to make sacrifices in terms of geography. I also think you need to weather rejection and stress well. |
| 863 | Throughly evaluate one's passion for science, that may come at the expense of lifestyle and income. |
| 864 | Throw yourself headfirst into your training and try to approximate, to the best of your ability, the lifestyle of someone in a research-intensive position. Make sure this is the life you want to live before you commit yourself to that career path. |
| 865 | To always think ahead to what type of career they want and to develop skills in line with that career |
| 866 | To be intrinsically motivated and resilient. Everyone's path is different; but if you stay focused and seek out mentors, the right path for you will be the one you are already on. |
| 867 | To be open mind with the opportunities, with the ideas and with life situations that not always goes in the way you maybe plan. |
| 868 | To be prepared to a log journey, one with no guarantee of a job at the end, and that includes sacrifice. That competition is high and the satisfaction of doing good science isn't always a guarantee. To make connections because they matter more than anything in finding work. To publish, and get grants. Apply for everything. |
| 869 | To carefully consider what that may look like (ie what their motivation is)- many people I have met do phds because they are waiting on medical school or don't know what to do. In my opinion, this would lead to poor satisfaction in a PhD |
| 870 | To choose a lab with both research quality and mentoring quality |
| 871 | To choose carefully in what lab spend their phd and to identify a mentor. |
| 872 | To consider each aspect of the job and the toll it takes before deciding if it is worth it. Furthermore to understand that this job is very different than a postdoc position and the qualifications required are mostly not taught during the entire scientific training period (from grad school through postdoc) |
| 873 | To definitely pursue it |
| 874 | To focus on doing good science, network as much as they can, and to read a lot. And try not fall into the current scientific enterprise in which publishing in high impact factor journal defines the quality of your research. And most important try to improve the scientific enterprise by making sure you make your science available to the public. |
| 875 | To follow what they are really passionate about. |
| 876 | To network and build collaborative relationship early, to keep an open mind, |
| 877 | To pursue experiences and activities that allow them to get a good insight into what the life of an academic is actually like and make a decision based on that |
| 878 | to really think about it, since when I think about an academic research, my raw model is Marie Curie, nowadays is not like that, more politics and big name, you can make it anyway, but the effort and the chances will not be proportional |
| 879 | To really think about what you want in life and how to achieve that. For me my career isn't first priority. To be very successful in an academic research career it helps if you put your career first. |
| 880 | To speak with a recent faculty hire about the prospects of an academic career. |
| 881 | To teach the younger student is one of the good reasons to choose academic research career. Moreover, you do not care about earning money for industry. You cam just enjoy your research. |
| 882 | To think about it very carefully, even though you will do what you love the field is not for faint hearted. Please be prepared for long days and no thank you's from your supervisor !!! |
| 883 | to think about the flexibility of being in academic research, as well as the time-consuming nature of research |
| 884 | To think about the path they have been on till now and how they see themselves 5 years from now |
| 885 | To think about why they want to go into a field in which only 5% of the people will get a position after years of sacrifices. |
| 886 | To try different area at least once (industry, policy, government, etc.) before you make a final choice. |
| 887 | To try long research internships before embarking on a PhD and be frank about the failures and work involved. |
| 888 | to understand the system, of publications and grants. Make sure that is what you want. |
| 889 | Transitioning into an academic research career is easier for people who work in translation/application oriented sciences than basic sciences, so try that. Try to get into using mice model systems from you PhD. Its easier to get grants. Choose a reputed lab because your PIs reputation in the field will take you a long way. Be ready to move to smaller universities to start your lab |
| 890 | Truly weigh the cost and benefits of the position. Then see if that is something you are interested in and think you are able to handle the challenges in that career. |
| 891 | Try a hand at research through research associate positions or short-term positions with academics, before plunging into a PhD program. Having a diverse portfolio helps, rather than one big project, one big book, or one big paper in the pipeline. I built my pipeline very slowly, and that gave me stress. I do think there is a need to step outside the publish or perish game, and craft one's own rules of engagement if possible. One way to do this is possibly doing work meaningful to oneself rather than looking at where the funding comes from, or what everyone else is doing. |
| 892 | try before you quit |
| 893 | Try not to limit yourself to one career trajectory. Be cognoscente that those who want you to succeed along a certain trajectory may gain indirectly by having you pursue that career trajectory. Take all advice on academic careers with a grain of salt --- survivorship bias runs rampant and otherwise scientific people often fail to see it in their own careers or in academia in general. |
| 894 | Try really hard, but don't stress yourself out too much to get there |
| 895 | try to balance your passion and your family, if you have one |
| 896 | Try to balance your personal life with your career choices |
| 897 | try to be productive |
| 898 | Try to be the more interdisciplinary you can. |
| 899 | Try to change the atmosphere from political and petty to collaborative and helpful. |
| 900 | Try to choose good mentors that keep you motivated while in training and let you pursue your interests. Work on topics that you find relevant that will also help you keep motivated. |
| 901 | Try to find a well funded lab whose PI is one of the top scientists in the field that you will work on. |
| 902 | Try to learn the more you can about every field because an interdisciplinary researcher has more chance of success. |
| 903 | Try to spend some time in a lab and work in a project before reaching a conclusion. |
| 904 | Try your best to do the experiment, submit paper, submit grant. You will never know what can happen to you. Cheers for my K99 award. |
| 905 | Understand and if possible experience the context, people, and politics before investing too much time and energy toward a potentially disenchanting career path |
| 906 | Understand and research all the available choices, as well as their pros and cons. |
| 907 | Understand that in the current funding and academic climate, family and other life priorities will take a back seat to your career if you want to be prestigious |
| 908 | Understand that it is a long road that is not necessarily always fair. Working hard does not equal success, unfortunately success if largely determined by who you know rather than hard work. |
| 909 | Understand the day to day tasks involved at PI level to see if this is something you like |
| 910 | Understand the full scope of academic positions. It's more than just research and teaching. Be open to assuming extra responsibilities. |
| 911 | Understand the path and limitations |
| 912 | Understanding that it is grant-based and the current probability of getting one. |
| 913 | Unless research is a burning desire, and you are consumed by the thirst for knowledge and research, DONT. |
| 914 | Unless you are in the top 10% and are willing to tip the work:life balance, don't bother. You also have to be well connected, have the right pedigree, and be lucky in addition to working hard. |
| 915 | Unless you are really dedicated and see academia as your only career goal, please see other options that are available out there. |
| 916 | Unless you are truly addicted to research and have the curious passion of a 3 years old, think twice before you make such a decision. |
| 917 | Unless you have rich parents and a lot of perseverance, think twice. |
| 918 | Unless you're really passionate about it, wait to see if the funding climate ever improves. |
| 919 | Very competitive but can be achieved with hard work and dedication. |
| 920 | very few faculty positions and a lot of postdocs... very competitive, a lot of effort and sacrifice in your everyday life, most likely you will have to move out of you country and you will not earn a lot of money |
| 921 | Very limited career opportunity beyond post-doc level position. |
| 922 | Vote. The US does not fund science at the levels necessary for everybody who wants to pursue an academic career to do so. |
| 923 | Weigh all the options, if discovery and freedom to do so is the best option for you then go for it. |
| 924 | Weigh the pros and cons. Know your strengths and weaknesses. Don't go into it for the money or easy life |
| 925 | Weight the pros and cons, and don;t expect making a living out of it |
| 926 | Well it is good to have academic career but personally i prefer to go for Industry based research work. |
| 927 | Work as a technician first and make sure that a Ph.D. is something you really want. Develop computational skills. Be sure you enjoy writing and giving presentations because your academic career depends on those two skills most of all. |
| 928 | Work hard and be nice to your colleagues. |
| 929 | Work hard and don't let your work be underestimated |
| 930 | Work hard and enjoy what you are researching |
| 931 | Work hard and never give up |
| 932 | Work hard and think hard. |
| 933 | Work hard and try to publish to high impact factor journal. Write grants/fellowship |
| 934 | Work hard and try your best. |
| 935 | Work hard with good ideas. |
| 936 | Work hard, be efficient and don´t be ashamed of your ideas and your work. Also, be very careful with the people/peers you meet in lab and science-not everyone is your friend-take care! |
| 937 | Work hard, evaluate the applicability of the science you want to make, ask your mentors to guide you. Write a lot of grants, produce a lot of data. Publish it! |
| 938 | Work hard, it is a long and tough way to follow if you want to succeed. |
| 939 | Work hard, read a lot, and always make questions. |
| 940 | Work hard, read papers, get scientific thinking |
| 941 | Work hard, sacrifice |
| 942 | Work hard, seize as many opportunities to attend conferences and give presentations, apply for grants, and publish. |
| 943 | Work hard, work on big problems, and choose the right mentors. |
| 944 | Work hard. Think about the practicality of what you are doing. Understand why you are doing every step that you are doing, if you do, you can build on that and improve the quality/speed of your given experiment. Think critically! |
| 945 | Work harder than everyone else, find good mentors, build a support system, and apply for everything. |
| 946 | Work in a lab. Attend lab meetings for different groups. Write a grant (F32/K99/R21). Read the literature. |
| 947 | Write. Write. Write. And then network! Network! Network. Communication and who you know are the most important keys to success in academic research. |
| 948 | You better have vocation |
| 949 | You can do it! |
| 950 | You can only do it if you love it, but it is the best job in the world |
| 951 | You can put in 80 hours a week, but unless you get lucky, you will not be able to publish in multiple high impact journals in order to attain a position in Academia. The hyper competitive climate in science is extremely discouraging to intelligent and innovative minds who can contribute tremendously to science and knowledge, but never got 'lucky' in their postdoc and therefore are discarded by Academia. How many past nobel laureates would have gotten tenure in today's Academia? How many brilliant breakthroughs will we miss with our current system of only hiring those who publish in high impact papers multiple times on 20-author projects? It's disturbing to think about. |
| 952 | You have to be committed to science, and understand that research is slow. |
| 953 | you have to be passionate |
| 954 | you have to be passionate about science to pursue it as a career |
| 955 | You have to be patient, persevering and deal well with frustration. You will not earn a lot of money, and you will have to work really hard all the time. |
| 956 | You have to decide if you are interested or passionate about research and some teaching. If one is just interested then its not the right career for them. |
| 957 | You have to do it because you are passionate and not think about high income |
| 958 | You have to focus on the sexy short term projects that can look flashy on the cover of a journal. Get as many publications out as quickly as possible regardless of scientific value or reproducibility. Game the author stats system because thats all that matters. Your name is a brand and if you succeed you can submit a napkin smeared with mustard to Nature and they will publish it. |
| 959 | You have to genuinely and intrinsically love this kind of work. If you have to constantly ask yourself if an academic research career is right for you, it probably is not. |
| 960 | You have to have your heart in it, and if you aren't interested in science outside of work it's probably not worth it |
| 961 | You have to love it to do it. |
| 962 | You have to love it... |
| 963 | You have to love research and discovery. |
| 964 | You have to love what you're doing or have a strong motivation to discover something otherwise you will probably burn out. You will fail sometimes but you just have to keep trying. Explore your options because despite what your mentors will tell you, there are other careers out there. |
| 965 | You have to really like science as you will be compromising in pretty much every other aspect of life (pay, job security etc) |
| 966 | You have to really love science to pursue a career in research. The stress can be overwhelming and the amount of effort you put in is not always rewarded. |
| 967 | You have to really want it. There is intense competition for few spots and you have to sacrifice a lot of other things in your life. Also, be realistic about how you want to play the game not just pure scientific ideals. |
| 968 | You have to think twice before going into this career path. |
| 969 | You must be passionate about your work, be willing to withstand a lot of failure and not give up. There is a good amount of luck associated with science but you must be prepared and hard working to reap the benefits of the luck. Be sure to explore other options as well as academic research because there are lots of other ways to have an exciting and fulfilling career. Also, talk to faculty at different levels in their careers and different types of institutions to determine if this might be a good career fit for you. |
| 970 | You must like adventure and snuggling is part of it, but it has its reward! |
| 971 | You must love what you do. |
| 972 | You must only begin a scientific career if you are passionate about the subject and not because of other external reasons such as money, prestige, etc. |
| 973 | you need perseverance and a strong emotional support group inside and outside academia. |
| 974 | You need to be okay with failures in day to day lab work. They happen from time to time, and there will be stretches without a success, but resilience is key to science. Also, always reach out for help from your colleagues. Science is meant to be a collaborative endeavor. |
| 975 | You need to be passionate about your project and have goals set to help yourself achieve those goals. |
| 976 | You need to be self-motivated and able to thrive in a field with little immediate gratification. Enter into it for the passion of wanting to know more, not to make money or prestige. |
| 977 | You need to genuinely love science and understand its not a guaranteed career |
| 978 | You need to really passionate about what you are doing. Otherwise, don't do science. |
| 979 | You need to recognize the challenges associate with it, especially financially. Can you live with a salary of 20k-30k as a graduate student until you are 30? Are you ready to start a family with a salary of 50k as a postdoc when your friends are making 70k-80k without a PhD? |
| 980 | You need to think twice before you enter an academic research career. Only if you like it from the bottom of your heart, then start one. |
| 981 | You really have to be passionate to be able to pursue. Learn to deal with frustration and failure. |
| 982 | You really have to love what you do and be self motivated to pursue and academic career |
| 983 | You really have to make sure that this is what you want to do because the salary is not matching the level of experience. And you have to be careful in the choices you make especially when you choose the lab you want to work in. Don't hesitate to make changes to reach what you aim for. |
| 984 | You really have to want it. Choose good mentors that publish well. Have a plan B career. |
| 985 | You really need to prepare yourself for failure. Most of your experiments won't work, reviewers (grants & papers) won't like what you're doing, etc. If you can still enjoy the research despite having the odds stacked against you, then pursue academic research as a career. |
| 986 | You should always apply for more grants than you need, and never hire a postdoc who's older than you are. |
| 987 | You should be excited about your research, or you're not doing it right. |
| 988 | You should consider that to be a good PI, you need not only research skills/ideas, but also personal skills to be successful in recruiting and mentoring people in your lab, and creating a good research environment. |
| 989 | You should pursue this path, only if you are very passionate about scientific discovery and like writing. |
| 990 | You will have to deal with constant pressure of getting funding so be prepared |
| 991 | You will need to be passionate about many different areas of research so that you can go where there's funding available but still be in a research area you love. |
| 992 | you won't make as much as you think you will, and you will work hard |
| 993 | Your heart has to be in it, you have to love research and problem solving and not care about financial gain. |
| 994 | Your primary focus should be on developing an expertise in obtaining grant funding. Get as much training, mentorship, and practice at selling your research to a funding agency. Of course, you also have to spend significant time on your research so that you have the raw materials for your grants. |
