# Supplementary material for: Postdocs’ advice on pursuing a research career in academia: A qualitative analysis of free-text survey responses
Source: PLoS One. 2021 May 6;16(5):e0250662. doi: 10.1371/journal.pone.0250662 (PMC8101926; doi:10.1371/journal.pone.0250662)
Supplement: S1 Table — (DOCX) [file pone.0250662.s002.docx]

| Code | Frequency |
| --- | --- |
| academia as a backup plan | 1 |
| academic freedom | 15 |
| academic life | 13 |
| administrative obligations | 7 |
| aptitude for academia | 5 |
| attend seminars | 1 |
| aware academia is a lifestyle | 9 |
| backup plan | 29 |
| be a good multi-tasker | 1 |
| be a perfectionist | 1 |
| be aware of immigration challenges | 1 |
| be aware of nepotism | 1 |
| be comfortable with environment | 6 |
| be comfortable with failure | 23 |
| be comfortable with uncertainty | 11 |
| be committed | 4 |
| be competitive | 4 |
| be creative | 4 |
| be curious | 10 |
| be determined | 11 |
| be efficient | 8 |
| be exceptional | 6 |
| be flexible | 12 |
| be focused | 11 |
| be idealistic | 1 |
| be innovative | 7 |
| be levelheaded | 1 |
| be motivated | 17 |
| be open-minded | 15 |
| be ready for difficult relationships | 6 |
| be ready for setbacks | 21 |
| be realistic about expectations | 29 |
| be resilient | 12 |
| be strategic | 19 |
| be tough | 2 |
| be versatile | 1 |
| be vigilant | 3 |
| boycott | 1 |
| broken system | 8 |
| burn out | 1 |
| career management | 1 |
| choose the right grad school | 3 |
| choose the right lab | 32 |
| collaborate | 21 |
| commit to doing good science | 4 |
| communication | 9 |
| community | 17 |
| connections | 13 |
| consider career alternatives | 47 |
| consider computer science | 1 |
| consider finance career | 2 |
| consider industry | 14 |
| consider science policy career | 1 |
| consider translational research | 2 |
| consider which aspects of research you like | 7 |
| cost-benefit ratio | 25 |
| cultivate scientific mind of the youth | 1 |
| dedication | 10 |
| demanding workload | 15 |
| different academic tracks | 2 |
| discipline | 3 |
| disproportionate distribution of grants to URM | 1 |
| don't go into academia | 56 |
| don't have to publish in top journals | 2 |
| don't lose track of time | 5 |
| don't overcommit | 1 |
| enjoy the journey | 13 |
| establish time frame | 9 |
| evaluate strengths and weaknesses | 4 |
| evaluate whether PhD is necessary | 1 |
| explore all options | 44 |
| explore current literature | 10 |
| explore your capabilities | 2 |
| family | 15 |
| famous lab | 8 |
| field in demand | 1 |
| field is competitive | 26 |
| financial stability | 48 |
| find your own niche | 4 |
| flexibility | 4 |
| focus on positives | 3 |
| frustrating | 10 |
| gain exposure as tech | 2 |
| gain exposure early | 14 |
| get MBA while postdoc | 1 |
| give presentations | 3 |
| go to medical school | 1 |
| goals | 18 |
| grantsmanship | 38 |
| hard work | 79 |
| have integrity | 2 |
| have multiple projects | 1 |
| have specific research focus | 1 |
| have thick skin | 8 |
| high quality research | 3 |
| higher degrees are a liability | 1 |
| holistic approach | 2 |
| if dream, it's worth it | 22 |
| imposter syndrome | 1 |
| informational interview | 14 |
| institutional support | 7 |
| job market | 14 |
| job opportunities | 9 |
| job requirements | 1 |
| know values | 27 |
| know your limitations | 1 |
| lab publication record | 2 |
| leadership | 3 |
| learn new skills | 1 |
| learn to ask for help | 1 |
| limited opportunities | 3 |
| location | 7 |
| long hours | 8 |
| long-term commitment | 41 |
| long-term gratification | 8 |
| luck | 30 |
| mental health | 5 |
| mentorship compatibility | 16 |
| meritocracy | 4 |
| money not primary motivation | 19 |
| motherhood | 1 |
| need for funding | 48 |
| need for publications | 38 |
| network | 35 |
| no room for doubts | 9 |
| non-bench careers | 1 |
| overworked | 3 |
| passion | 189 |
| patience | 26 |
| perseverance | 45 |
| personal brand | 3 |
| PI Job description | 7 |
| plan ahead | 17 |
| politics | 15 |
| poor lab training | 1 |
| postpone commitment | 1 |
| power structure | 2 |
| publish in high impact journals | 14 |
| purpose | 23 |
| pursue hobbies first | 1 |
| pursue masters | 1 |
| qualifications | 5 |
| quality of life | 3 |
| reassurance | 7 |
| recognition | 6 |
| research before committing | 42 |
| revise plan regularly | 3 |
| rewarding | 25 |
| sacrifice | 37 |
| saturated field | 19 |
| self-attained success | 1 |
| self-confidence | 19 |
| self-learn | 4 |
| self-reflect | 75 |
| self-worth | 5 |
| start simple | 1 |
| strengthen writing skills | 4 |
| strengthen research skills | 12 |
| strong mentorship | 58 |
| strong scientific base | 1 |
| success not guaranteed | 27 |
| survivorship bias | 2 |
| take time off | 2 |
| talk to people in various stages | 15 |
| teach | 4 |
| tedious | 1 |
| temporary position | 1 |
| transferable skills | 15 |
| trust your instincts | 1 |
| understand the risk | 12 |
| utilize your advantages | 1 |
| well-established PI | 12 |
| wellness | 5 |
| work as intern | 2 |
| work environment | 3 |
| work on noteworthy problems | 1 |
| work-life balance | 32 |
