# Supplementary material for: Postdocs’ advice on pursuing a research career in academia: A qualitative analysis of free-text survey responses
Source: PLoS One. 2021 May 6;16(5):e0250662. doi: 10.1371/journal.pone.0250662 (PMC8101926; doi:10.1371/journal.pone.0250662)
Supplement: S2 Table — (DOCX) [file pone.0250662.s003.docx]

| **Category** | **Codes** | | | | |
| --- | --- | --- | --- | --- | --- |
| Academic life | Academic freedom | Academic life | Administrative obligations | Aware academia is a lifestyle | Grantsmanship |
|  | Leadership | Meritocracy | Need for publications | Sacrifice | Teach |
| Challenges | Be aware of immigration challenges | Be aware of nepotism | Be comfortable with failure | Be ready for difficult relationships | Burn out |
|  | Demanding workload | Disproportionate distribution of grants to URM | Field is competitive | Frustrating | Hard work |
|  | Higher degrees are a liability | Limited opportunities | Long hours | Luck | Overworked |
|  | Politics | Power structure | Sacrifice | Saturated field | Success not guaranteed |
|  | Tedious |  |  |  |  |
| Financial security | Financial stability | Money not primary motivation | Need for funding | Sacrifice |  |
| Understand the risk | Be comfortable with failure | Be ready for setbacks | Cost-benefit ratio | Luck | Sacrifice |
|  | Understand the risk | Temporary position |  |  |  |
| Wellness | Mental health | Quality of life | Self-confidence | Self-worth | Take time off |
|  | Wellness |  |  |  |  |
| Work-life balance | Demanding workload | Family | Motherhood | Sacrifice | Work-life balance |
