# Supplementary material for: Postdocs’ advice on pursuing a research career in academia: A qualitative analysis of free-text survey responses
Source: PLoS One. 2021 May 6;16(5):e0250662. doi: 10.1371/journal.pone.0250662 (PMC8101926; doi:10.1371/journal.pone.0250662)
Supplement: S3 Table — (DOCX) [file pone.0250662.s004.docx]

| **Category** | **Codes** | | | | |
| --- | --- | --- | --- | --- | --- |
| Career planning | Academia as a backup plan | Backup plan | Be comfortable with environment | Be realistic about expectations | Career management |
|  | Consider career alternatives | Consider which aspects of research you like | Different academic tracks | Evaluate whether PhD is necessary | Explore all options |
|  | Find your own niche | Flexibility | Gain exposure as tech | Gain exposure early | Get MBA while postdoc |
|  | Informational interview | Institutional support | Job market | Job opportunities | Job requirements |
|  | Location | Open-minded | PI Job description | Plan ahead | Postpone commitment |
|  | Pursue hobbies first | Research before committing | Revise plan regularly | Survivorship bias | Take time off |
|  | Talk to people in various stages | Work environment | Work as intern |  |  |
| Non-academic careers | Backup plan | Consider career alternatives | Consider computer science | Consider finance career | Consider industry |
|  | Consider science policy career | Consider translational research | Explore all options | Go to medical school | Non-bench careers |
|  | Pursue masters |  |  |  |  |
| Commitment | Be flexible | Dedication | Demanding workload | Discipline | Don’t lose track of time |
|  | Don't overcommit | Enjoy the journey | Establish time frame | Long hours | Long-term commitment |
|  | Long-term gratification | No room for doubts | Perseverance | Sacrifice | Self-attained success |
| Don't go into academia | Boycott | Broken system | Don't go into academia |  |  |
| Laboratory | Choose the right laboratory | Famous laboratory | Laboratory publication record | Poor laboratory training | Well-established PI |
|  | Work environment |  |  |  |  |
| Mentorship | Learn to ask for help | Mentorship compatibility | Strong mentorship | Well-established PI |  |
| Network | Collaborate | Communication | Community | Connections | Network |
| Passion | If dream, it’s worth it | Long-term gratification | Money not primary motivation | Passion | Rewarding |
|  | Sacrifice |  |  |  |  |
| Publications | Don’t have to publish in top journals | Need for publications | Publish in high impact journals | Publish in small journals |  |
| Purpose | Goals | Purpose | Recognition | Rewarding |  |
| Qualities of a good scientist | Aptitude for academia | Be comfortable with failure | Be comfortable with uncertainty | Be creative | Be curious |
|  | Be determined | Be a good multi-tasker | Be a perfectionist | Be competitive | Be efficient |
|  | Be exceptional | Be flexible | Be focused | Be idealistic | Be innovative |
|  | Be level-headed | Be motivated | Be open-minded | Be resilient | Be strategic |
|  | Be tough | Be versatile | Be vigilant | Cultivate scientific mind of the youth | Dedication |
|  | Discipline | Explore your capabilities | Have integrity | Have thick skin | Leadership |
|  | Patience | Perseverance | Self-confidence | Strong scientific base |  |
| Research skills | Commit to doing good science | Explore current literature | Grantsmanship | High quality research | Strengthen research skills |
|  | Strengthen writing skills | Strong scientific base |  |  |  |
| Self-reflection | Consider which aspects of research you like | Explore your capabilities | Imposter syndrome | Know values | Know your limitations |
|  | Qualifications | Reassurance | Self-reflect | Evaluate strengths and weaknesses |  |
| Strategy | Attend seminars | Be competitive | Be efficient | Be exceptional | Be flexible |
|  | Be strategic | Choose the right grad school | Choose the right laboratory | Don't overcommit | Explore current literature |
|  | Famous laboratory | Field in demand | Focus on positives | Get MBA while postdoc | Give presentations |
|  | Have multiple projects | Have specific research focus | Holistic approach | Learn new skills | Learn to ask for help |
|  | Personal brand | Self-learn | Start simple | Utilize your advantages | Well-established PI |
|  | Take time off | Trust your instincts | Work on noteworthy problems |  |  |
| Transferable skills | Explore your capabilities | Learn new skills | Transferable skills |  |  |
