# Supplementary material for: Postdocs’ advice on pursuing a research career in academia: A qualitative analysis of free-text survey responses
Source: PLoS One. 2021 May 6;16(5):e0250662. doi: 10.1371/journal.pone.0250662 (PMC8101926; doi:10.1371/journal.pone.0250662)
Supplement: Attachment — Submitted filename: PONE-D-20-40531 review.docx [file pone.0250662.s005.docx]

This is a timely article that reflects the attitude of postdocs towards pursuing careers in academia in research-intensive institutions as tenure/tenure-track faculty. The open ended free text responses on guidance/advice to graduate students and undergraduates interested in pursuing careers in the biological sciences will certainly be useful as they consider the next steps in their training trajectories.

There are a few questions that would be helpful to address in this article:

1. While I understand that the authors can simply not survey all 21,781 NSF-identified postdocs in the U.S., it is important to explain on what basis the 1,248 survey recipients were selected since this is a very small percentage of total postdocs. What constitutes “top” institutions? On what criteria are those determinations based? How many institutions were included? Are the rankings based on NIH funding, US News and World Report? What is the geographic distribution? How many are public versus private institutions? AAU institutions? Perhaps even naming the institutions would be useful.
2. Were the postdocs primarily in schools or colleges of medicine?
3. What percent of respondents had originally planned to pursue academic careers and how many subsequently changed their minds?
4. How many respondents had any type of individual extramural fellowships (such as the F kiosk from the NIH)
5. Is the use of the IDP mandatory at these institutions? How many respondents actually availed themselves of an IDP to hold discussions regarding career choices with their research mentors?
6. What is the faculty perception among mentors of the postdocs surveyed about their attitudes? Was that even asked in the survey? Even if the question was not asked in the survey, a discussion of the literature around faculty perceptions in the Discussion would be helpful.
