# Supplementary material for: Postdocs’ advice on pursuing a research career in academia: A qualitative analysis of free-text survey responses
Source: PLoS One. 2021 May 6;16(5):e0250662. doi: 10.1371/journal.pone.0250662 (PMC8101926; doi:10.1371/journal.pone.0250662)
Supplement: Attachment — Submitted filename: PLOS ONE rebuttalletter 4-4-21.docx [file pone.0250662.s006.docx]

W. Marcus Lambert, PhD, MS

SUNY Downstate Health Sciences University

450 Clarkson Ave.

Brooklyn, NY 11203

Frederick Grinnell, PhD

Academic Editor

PLOS ONE

April 2, 2021

Dear Dr. Grinnell and Reviewers,

Thank you for your very thoughtful review of the manuscript (PONE-D-20-27016): *“****Postdocs’ advice on pursuing a research career in academia: A qualitative analysis of free-text survey responses****”* by Suwaiba Afonja, Damonie G. Salmon, Shadelia I. Quailey, and W. Marcus Lambert (corresponding author) for consideration as a research article in *PLOS ONE*. We would like to offer the following modifications and comments based on your review. Please see below:

**Reviewer #1:**

1. While I understand that the authors can simply not survey all 21,781 NSF-identified postdocs in the U.S., it is important to explain on what basis the 1,248 survey recipients were selected since this is a very small percentage of total postdocs. What constitutes “top” institutions? On what criteria are those determinations based? How many institutions were included? Are the rankings based on NIH funding, US News and World Report? What is the geographic distribution? How many are public versus private institutions? AAU institutions? Perhaps even naming the institutions would be useful.

All survey respondents self-selected to participate in the survey based on the inclusion and exclusion criteria previously published. The sample represents wide geographic (over 80 universities) and subfield diversity. The number of institutions and the percentage of respondents from each institution were published as a supplementary file (suppl file 2) here: <https://cdn.elifesciences.org/articles/48774/elife-48774-supp2-v2.xlsx>. We also determined the percentage of respondents from highly-ranked life science research institutions in the US based on counts of high-quality research outputs between January 1, 2017 and December 31, 2017 according to rankings from *Nature Index* (Figure 1- figure supplement 1D, <https://elifesciences.org/articles/48774#fig1s1>). As noted in our previously published work, the majority of respondents are from highly ranked US institutions, but the differences in institutional ranking do not fully account for the differences in career intention. We have included this information now in Methods (lines 220 – 234).

2. Were the postdocs primarily in schools or colleges of medicine?

About 40% of the postdocs were not affiliated with a hospital, medical center, or college of medicine.

3. What percent of respondents had originally planned to pursue academic careers and how many subsequently changed their minds?

60% of postdoc respondents were less committed to an academic career since starting their postdoctoral position.

4. How many respondents had any type of individual extramural fellowships (such as the F kiosk from the NIH)

We did not ask about extramural fellowships, and the dataset is anonymous.

5. Is the use of the IDP mandatory at these institutions? How many respondents actually availed themselves of an IDP to hold discussions regarding career choices with their research mentors?

The NIH strongly encourages individual development plans (IDPs) for all NIH-supported graduate students and postdoctoral researchers, whether on training grants, fellowships, or research project grants. Those who are on these grants must report on the use of IDPs in their Research Performance Progress Report (RPPR). While we didn’t ask postdocs about the use of IDPs, we asked: How would you rate your understanding of the careers available to you? 94% of respondents said either “significant” (39%) or “moderate” (55%).

6. What is the faculty perception among mentors of the postdocs surveyed about their attitudes? Was that even asked in the survey? Even if the question was not asked in the survey, a discussion of the literature around faculty perceptions in the Discussion would be helpful.

We did not ask the mentors about postdoc attitudes, however, there is a really good paper by seven institutions that hold Broadening Experiences in Scientific Training (BEST) programs in *PLOS One*. They conducted faculty surveys of their BEST mentors to understand faculty perceptions around career development for their trainees. The faculty believed that there was a shortage of tenure track positions and felt a sense of urgency in introducing broad career activities for their trainees. They also found that faculty perceived trainees themselves as lacking in the knowledge base of skills that are of interest to non-academic employers. We believe this further highlights the need for this publication, and we cite this paper now in the discussion (lines 780 – 788).

**Reviewer #2:**

1. The methodology employed is not grounded theory. Clearly both the author’s previous work and prior studies framed what they expected to see. Thus, it could not be a neutral, outcome-agnostic approach required of grounded theory. It is content or thematic analysis.

The objective of this work is to share postdoctoral researchers’ advice on pursuing an academic research career. In response to the open-ended question posed, the postdocs provided recommendations based on their personal experiences. Upon review, we agree that while our approach sought to remain neutral, our previous study (survey) framed the context for this question. However, we used systematic qualitative methods to ensure objectivity of responses and framed the question in this study to be open-ended and objective. We built upon the previous study using hallmarks of both content analysis (examining patterns in text, highlighting frequency counts) and thematic analysis (interpreting themes within the data). We’ve updated the text to reflect this (lines: 28 and 196 -197).

2. How the many nodes are combined into the 20 themes they refer to is never presented. Without that, a reader has no idea how many and which of the nodes contributed to the 2 themes they report on here.

We have attached Supporting Information files that show how the 177 nodes were divided into 20 categories, with some overlap, under 10 subthemes (Academia is a lifestyle, Postdocs feel underpaid and undervalued, Prepare for multiple career paths, Reflect on your motivation, Assess readiness, Be strategic, Choose the right laboratory, Choose mentors wisely, Publication strategies to consider, and Network) and two thematic areas (Life in Academia and Strategies for Success).

3. What were the other themes and why are they not reported on as well?

Many of the categories that were derived from the data were organized into 10 subthemes (bolded subheadings throughout the manuscript) that we recognized could be further organized into 2 major thematic areas. We chose these broad areas to help the readers (who we expect to be a very diverse audience) understand and follow the paper.

4. A major limitation of the study is that, as stated in lines 170-171, they only offered the survey to “postdoctoral listservs from top-ranked research universities and institutions”. Thus, by definition, their data to not speak to perceptions and advice of the very many postdocs at other universities. This is a significant limitation that should be noted in the methods as well as in the limitations section so that that readers are fully informed as they consider the data presented. Arguably, the perceptions and advice are contextualized by the extreme research competitiveness and high expectations at these institutions.

As noted in response to Reviewer #1, the number of institutions and the percentage of respondents from each institution were published as a supplementary file (suppl. file 2) here: (<https://cdn.elifesciences.org/articles/48774/elife-48774-supp2-v2.xlsx>). We also determined the percentage of respondents from highly-ranked life science research institutions in the US based on counts of high-quality research outputs between January 1, 2017 and December 31, 2017 according to rankings from *Nature Index* (Figure 1- figure supplement 1D, <https://elifesciences.org/articles/48774#fig1s1>). As noted in our previously published work, the majority of survey respondents are from highly ranked US institutions, but the differences in institutional ranking do not fully account for the differences in career intention. We have included this information now in Methods (lines 220 – 234).

We agree this is a limitation of the study and have indicated this in the methods and limitations section (lines: 814 – 820). However, it’s important to note that postdoctoral appointees at the top 100 institutions in the country (n=56,092) account for approximately 88% of the total number of postdocs in the United States (n=63,861). Therefore, our sample is representative of a significant portion of the U.S. postdoc population.

5. With such a large fraction of their respondents being international postdocs, it is important that they provide data comparing their responses to non-international postdocs. Evidence of the significance of this population is provided by the average time to the PhD of 4.6 years, which is well-below that for U.S. PhD programs. This likely reflects the shorter, time-constrained PhD training in many countries outside the U.S.

The large population of international postdocs in this study may account for the decreased average time to the PhD reported in the manuscript but we did not find significant differences in their responses across immigration status. At the time of the study, all of the respondents have had at least one postdoctoral appointment in the United States and provide similar recommendations both in support of and against pursuing an academic research career independent of domestic or international status. We have made note of the difference in time to the PhD in the Results at lines: 256-258.

6. It also would be very helpful to know how men and women respond the same or differently. Comparison with UR postdocs would be valuable as well since their numbers are high at 174.

The postdocs responded similarly across gender, race/ethnicity, and national status. We have noted this on lines: 253-254.

7. It is not clear how the background on postdocs support (intro paragraph 2 and data in results) are germane to the purpose and results of the study. What is presented is true but common knowledge and the results reported don’t add any new insights into this topic.

Financial insecurity was among the most referenced challenges in the postdocs’ responses. The codes “financial stability” and “need for funding” were both cited 48 times. We introduced and discussed financial support because the postdocs reported it to be a major concern. We believe that this topic would be especially useful for career preparation for underrepresented minority trainees and first-generation students and scientists who may not be familiar with such information. Moreover, no other published report that we can find has discovered what our work shows both quantitatively and qualitatively: postdocs are not simply having financial challenges, but for many, a perceived sense of financial insecurity can result in a career change.

8. In lines 97-99 the authors state: “This loss of interest may be remedied by an increase in transparency about life in academia and disseminating more information about the critical steps for securing an academic research position [31]”. It is unclear where this statement is coming from as it is counter-intuitive given the assertions leading up to it re: exit from academic careers interests. It would seem that more information about the challenges could only lead to more loss of interest.

In the current literature, there have been numerous calls for increased transparency on career paths beyond the PhD. Since 2018, a number of schools through the Coalition for Next Generation Life Sciences have been releasing career outcomes and admissions data on their schools. This has not seemed to affect admissions to these institutions. On the contrary, open data and transparency about the positives and challenges of an academic career would allow for a more informed decision, perhaps earlier in the training path. There is not much formal documentation on life in academia from the perspective of postdocs, so we address this gap in the literature.

Our previous work shows that there is a loss of interest in academic research careers as the postdoctoral training period progresses with a sharp regain of interest in the latter years. We found from our previous survey that many of the postdoctoral trainees are seeking more transparency around how to succeed in an academic career. More than 65% of trainees noted that a transitioning to research independence course would increase their likelihood of pursuing a research career in academia. Our data shows that many would rather faculty be forthcoming about the good, bad, and ugly. We believe this clarity earlier on will not ultimately result in a loss of postdocs who apply for faculty positions, rather save many trainees the loss of time in discovering how their interests fit with their career goals.

9. There was good effort to provide counter-balancing quotations around concerns expressed by any 1 individual with quotations where those concerns were either not seen as challenges or were seen as manageable by others. Any revisions to the MS should continue to provide counter-balancing examples to avoid leading a reader to perceive any single quote as representing ‘truth’ vs. a statement by 1 individual.

We tried very hard to support inferences from the text with balanced and representative quotes. Our revisions will continue to provide counter-balancing examples.

10. Results starting on line 292 don’t seem to be germane to this paper and represent the same findings that many other studies have reported previously.

See our response to comment #7. These challenges were some of the most cited in the text. Again, no other publications have found that postdocs’ financial insecurity are indeed impacting career decisions.

11. Staring on line 316 it appears that statements with respect to professional development support will be forthcoming but those do not materialize. If they want to devote a section to professional development resources it would need to balance those who feel they have adequate support and those who do not. However, this highlights one of the SIGNIFICANT limitations of a single item survey study – with such a broad array of what someone might choose to comment on, there is no way to draw conclusions on importance of peripheral topics such as this. Analytically, one is limited by what the question triggers a respondent to think about, and, for example, career development support would not be a likely topic of primary focus.

The reviewer is correct about the limitations of using a single item survey which we also mention in our limitations section (lines 805 – 807). We would also like to note that the “Strategies for Success” section should help interested readers with their career planning and development.

12. The results on suggestions for those considering academic careers is by far the most important and useful contribution of this study. It is generally well laid out and could be valuable for PhD students and postdocs. Revisions to the MS should keep that in mind so its centrality is not lost.

We thank the reviewer for this feedback.

13. The Discussion does not significantly speak to how the findings of this study compare to those of previous studies, and what is new about their findings.

Based on comments 7 and 8, we have revised our discussion accordingly. As noted before, there is little published data from postdoctoral fellows demonstrating the impact of these perceived challenges on career choice. *Nature* has conducted a study recently, but it is not author-driven. Understanding the perspectives of postdoctoral trainees and what diverts them from research careers will help increase persistence in the scientific workforce.

14. In lines 611-612 the authors state: “We also suspect that not everyone considers the financial impact of years of training until they are immersed in it.” This seems to be conjecture and no data supporting it come from the study. The follow-on sentence about postdoc frustrations is certainly not new information.

Regarding our statement about the financial impact of training: This is inferred from several comments on finances during the training period. One quote (which we did not put in the manuscript, but is available in the supplementary files) said, “…look for a financially stable partner, who has a real job, so that you can continue your fake postdoc job.” Another said, “Be prepared to give up your life and finances for the next 5-10 years.” Another noted, “If you want financial security sooner than later, I would avoid science at least jobs in academia.” These trainees are giving advice about financial considerations because it has become more of a factor in their career decisions now. While postdoc salaries are not completely new information, there are no reports that we are aware of that report on this perception from the postdoctoral perspective. This qualitative data shows the extent to which postdocs feel that it has impacted their career choice.

15. Starting on line 631, the paragraph speaks to frustrations with the job market for academic careers, which again is not new information. The authors go on to say, however: “Their responses seem to signal a shift in academia that is underscored by hypercompetitive climates, poor financial compensation, and a shrink in tenure-track faculty positions.” It is not clear why they say this is ‘evidence’ of a shift which most would say has been occurring over the past decades. Furthermore, it is not clear that the number of tenure track positions is declining. Most reports point to how the number of TT positions is flat and all of the growth in academic positions as been in non-TT positions. If there is evidence to support their conclusions it should be referenced.

We have modified this section to suggest that our data confirms previously published sentiments that the job market has become more competitive. The Reviewer 2 is correct in that the number of tenure-track positions is flat, not declining. We have updated the text to clarify that we meant there is a shortage in the number of *available* tenure-track positions as the number of postdocs have more than tripled over the past few decades, among other reasons (lines: 74 – 78). We have also updated the text to note data showing that only 15% of postdocs secure tenure-track position and that faculty feel they do not have the necessary skills and resources to support the remaining 85% (lines: 717 – 719).

16. In lines 638-640, the authors conclude: “The crux of this statement suggests that only under perfect conditions will postdocs make it through the academic pipeline.” This is not a conclusion that can be reasonably drawn from the quote upon which it is based. In particular the setting out of ‘perfect conditions’ is not justified from the data provided.

Thank you for this assessment. We have removed this statement.

17. Under study limitations, it is important to point out that while the single very open question allows for a wide range of responses, it means that any responses that are very infrequent and/or tangential to what most choose to comment upon must be considered very cautiously. This is true to such a point that they should not be included as quotes in the MS as representative if they come from minor nodes. Since it is not clear which nodes quotes are drawn from, a reader cannot assess if this is true or not.

We have updated the text to make note of this limitation (lines: ). We have also updated our Supporting Information files to include a table (S1_Table) of the original codes with their corresponding frequencies and two additional tables (S2_Table and S3_Table) showing how the 177 nodes were divided into 20 categories, with some overlap, under 10 subthemes (referenced above) and two thematic areas (Life in Academia and Strategies for Success). With this information, readers can recognize the major and minor codes by examining the provided frequencies and then observing how the codes were arranged into the 20 categories that the 10 subthemes were derived from.

Sincerely,

W. Marcus Lambert, PhD, MS
